# Accuracy and Completeness of Drug Information in Wikipedia: A Comparison with Standard Textbooks of Pharmacology

**DOI:** 10.1371/journal.pone.0106930

**Published:** 2014-09-24

**Authors:** Jona Kräenbring, Tika Monzon Penza, Joanna Gutmann, Susanne Muehlich, Oliver Zolk, Leszek Wojnowski, Renke Maas, Stefan Engelhardt, Antonio Sarikas

**Affiliations:** 1 Institute of Pharmacology and Toxicology, Technische Universität München, Munich, Bavaria, Germany; 2 Walther-Straub-Institute of Pharmacology and Toxicology, Ludwig-Maximilian-University, Munich, Bavaria, Germany; 3 Institute of Pharmacology of Natural Products & Clinical Pharmacology, University of Ulm, Ulm, Baden-Württemberg, Germany; 4 Department of Pharmacology, University Medical Centre of Johannes Gutenberg University, Mainz, Rhineland-Palatinate, Germany; 5 Institute of Experimental and Clinical Pharmacology and Toxicology, Friedrich-Alexander University Erlangen-Nürnberg, Erlangen, Bavaria, Germany; University Hospitals of Geneva, Switzerland

## Abstract

The online resource Wikipedia is increasingly used by students for knowledge acquisition and learning. However, the lack of a formal editorial review and the heterogeneous expertise of contributors often results in skepticism by educators whether Wikipedia should be recommended to students as an information source. In this study we systematically analyzed the accuracy and completeness of drug information in the German and English language versions of Wikipedia in comparison to standard textbooks of pharmacology. In addition, references, revision history and readability were evaluated. Analysis of readability was performed using the Amstad readability index and the Erste Wiener Sachtextformel. The data on indication, mechanism of action, pharmacokinetics, adverse effects and contraindications for 100 curricular drugs were retrieved from standard German textbooks of general pharmacology and compared with the corresponding articles in the German language version of Wikipedia. Quantitative analysis revealed that accuracy of drug information in Wikipedia was 99.7%±0.2% when compared to the textbook data. The overall completeness of drug information in Wikipedia was 83.8±1.5% (p<0.001). Completeness varied in-between categories, and was lowest in the category “pharmacokinetics” (68.0%±4.2%; p<0.001) and highest in the category “indication” (91.3%±2.0%) when compared to the textbook data overlap. Similar results were obtained for the English language version of Wikipedia. Of the drug information missing in Wikipedia, 62.5% was rated as didactically non-relevant in a qualitative re-evaluation study. Drug articles in Wikipedia had an average of 14.6±1.6 references and 262.8±37.4 edits performed by 142.7±17.6 editors. Both Wikipedia and textbooks samples had comparable, low readability. Our study suggests that Wikipedia is an accurate and comprehensive source of drug-related information for undergraduate medical education.

## Introduction

The omnipresence of the internet has an increasing impact on higher education and on the way students access information for learning. The current generation of undergraduate students grew up in an environment in which the internet and computer-based technologies have become an integral part of life. This cohort, often referred to as “Net Generation” or “Digital Natives” is technological savvy and familiar with the world wide web as a communication platform and source of information [1]. In addition, studies have suggested differences in learning behavior and learning preferences of “Digital Natives” in comparison to preceding student generations. For instance, Oblinger and colleagues proposed that today's students are more comfortable with multimedia learning environments, prefer to be actively engaged rather than being passive consumers of information and favor immediate responses and topic-related discussions with their peers and educators, both in person and online [2].

In parallel, the world wide web has advanced from a network of static web sites to the more user-centered “Web 2.0” that supports active participation, dynamic interaction and collaborative approaches [3]. One key element of the “Web 2.0” are wikis (a term originating from the Hawaiian word for “quick”) which are web applications that allow users to collaboratively create, edit and share content. Wikis have little implicit structure and thus allow content to emerge according to the needs of the users. The most frequented wiki of the internet is the online encyclopedia Wikipedia (www.wikipedia.org). The English language version of Wikipedia (www.en.wikipedia.org) currently consists of 4.54 million articles (yearly change +8.0% or 362,933 articles) written by approx. 40,000 registered editors. Each month, the website has more than 9.5 billion page views and 2.9 million article revisions (http://stats.wikimedia.org; accessed 18. April 2014). The smaller German language edition of Wikipedia (www.de.wikipedia.org) consists of approx. 1.67 million articles (yearly change +8.0% or 133,526 articles) created by approx. 7,900 registered editors. It has approx. 1.27 billion page views and 0.6 million article revisions per month (http://stats.wikimedia.org; accessed 18. April 2014). Since its launch in 2001, Wikipedia has become one of the most visited websites and a frequently accessed source of medical information [4]. Recent studies estimated that Wikipedia is used by up to 70% of junior physicians and practitioners as information source for medical care [5]. Moreover, Wikipedia has been shown to be widely used by undergraduate medical students [6], [7].

In contrast to traditional encyclopedias that are written and edited exclusively by professionals, Wikipedia allows any user to edit content and create articles. This lack of a formal editorial review and the heterogeneous expertise of contributors often results in skepticism by both educators and students whether Wikipedia should be recommended as an information source in undergraduate medical education [8].

While a number of empirical studies has evaluated the medical content of Wikipedia (reviewed in [9]), only few researchers have addressed the quality of pharmacological information. Of these, research has focused on a limited set of drugs or on information for patients and health practitioners [10], [11]. Whether Wikipedia is a suitable source of pharmacological information for students and educators in medical education has not been investigated.

In this study we provide a comprehensive analysis of accuracy and completeness of drug information in Wikipedia in comparison to standard textbooks of basic pharmacology. In addition, references, revision history and readability of the corresponding drug articles in Wikipedia were analyzed to evaluate their potential as a learning resource in undergraduate medical education.

## Methods

### Study design

**Figure 1:**
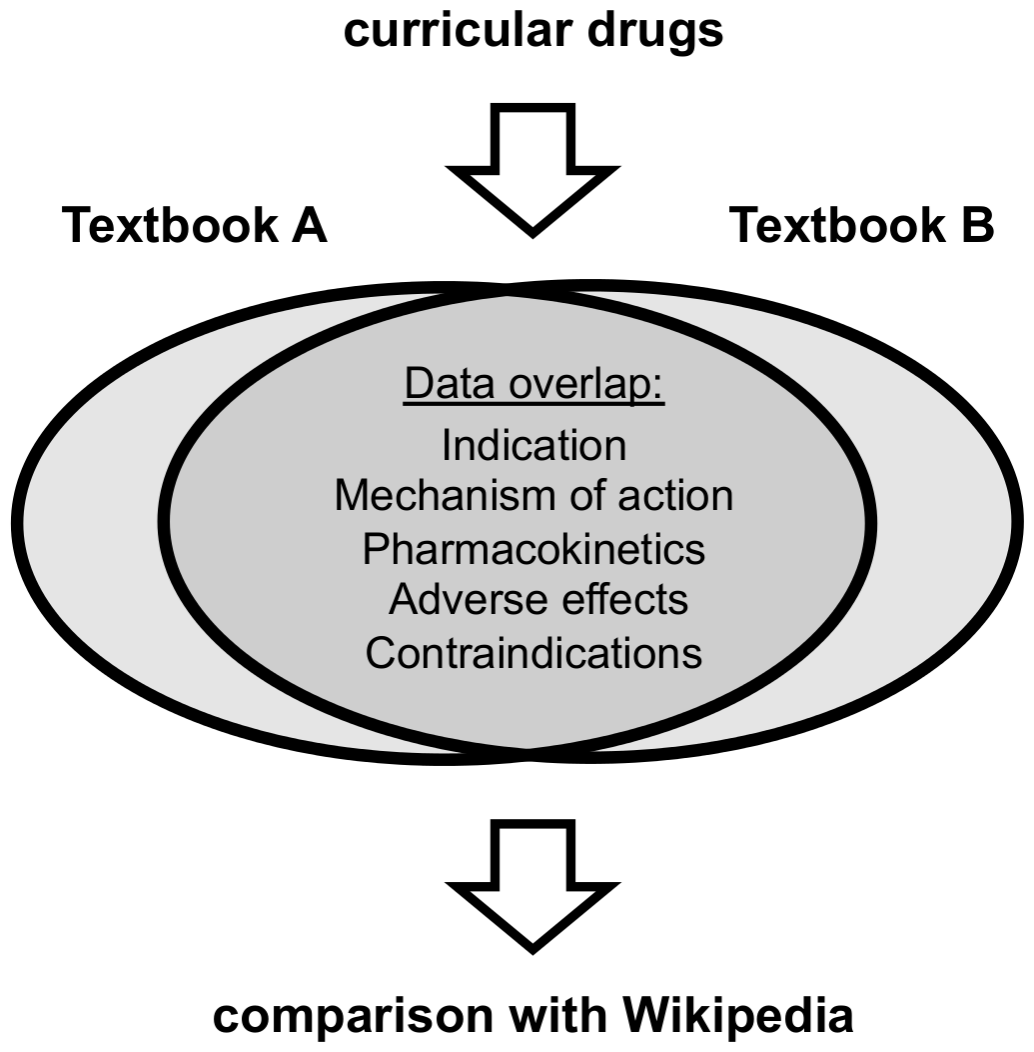
Study design. Pharmacological information of randomly selected curricular drugs was retrieved form two standard textbooks of general pharmacology for five different categories (indication, mechanism of action, adverse effects, pharmacokinetics and contraindications). The textbook data overlap served as point of reference for the corresponding drug articles in Wikipedia. The analysis was performed separately for the German and English language version of Wikipedia and textbooks.

One hundred drugs were randomly selected from the list of 300 curricular drugs (www.tum300.de) taught in a basic pharmacology course for undergraduate medical students in Munich (see **table S1** for the list of selected drugs). The TUM300 drug list is based on the World Health Organization Model Lists of Essential Medicines and adjusted to include all major pharmacotherapeutic substance classes and drugs that are most commonly prescribed in Germany [12]. For each drug, information on indication (IND), mechanism of action (MA), adverse effects (AE), pharmacokinetics (PK) and contraindications (CI) was excerpted from two German standard textbooks of basic pharmacology [13], [14]. Information on drug-drug interactions was included in the “pharmacokinetics” category. Only information present in both textbooks (data overlap) was included in the study and served as point of reference for the analysis of the respective drug articles in the German language version of Wikipedia (www.de.wikipedia.org; last accessed on March 20th 2013) (**Figure 1**). An analogous approach was used to compare the data overlap of 50 drugs (see **table S1**) retrieved from two standard Anglo-American textbooks [15], [16] with the corresponding drug articles in the English language version of Wikipedia (www.en.wikipedia.org; last accessed on June 20th 2014).

### Assessment of accuracy and completeness

Accuracy was defined as the degree of closeness to the pharmacological information of the textbooks overlap. Accuracy of information was evaluated separately for each drug and category and quantified by calculating the number of correct statements in Wikipedia divided by the total number of statements in the textbook overlap.

Completeness was defined and calculated as percentage of pharmacological statements of the textbook overlap present in the respective Wikipedia articles. All statements missing in Wikipedia were re-assessed for didactic relevance in the undergraduate medical education of basic pharmacology by six lecturers of pharmacology (teaching experience >5 years) of five different universities in Germany. Didactic relevance was defined as information that is essential for the understanding of pharmacokinetic and pharmacodynamics principles and characteristics of drugs as outlined in the German Medical Licensure Act (Approbationsordnung für Ärzte) [17]. A statement was considered relevant if more than four out of six raters endorsed its relevance for teaching.

### Assessment of references

For each Wikipedia article all references were downloaded (www.de.wikipedia.org; accessed on March 20th 2013; www.en.wikipedia.org; accessed on November 15th 2013) and grouped in the following categories: “academic journals” (publications in peer-reviewed academic journals), “scientific databases and drug info” (official drug databases and prescribing information), “textbooks”, “professional organizations”, “news media” (print or web), “commercial companies” (non-peer reviewed drug information provided by pharmaceutical companies). The total number and reference categories for all drug articles evaluated were pooled and descriptive statistical analysis performed using GraphPad PRISM 5.0 (La Jolla, CA).

### Number of revisions and editors

Data on the total number of edits and editors (registered, anonymous and automated “bot” editors) for each drug article in the German language version of Wikipedia was provided by Wikimedia Foundation (San Francisco, CA). Descriptive statistical analysis was performed using GraphPad PRISM 5.0 (La Jolla, CA) software.

### Assessment of readability

Readability was evaluated using the Amstad readability index, a variant of the Flesch Reading Ease Sore adapted to the analysis of German texts [18]. The Amstad index (*R_Amstad_*) is based on the equation 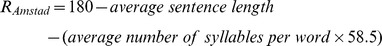



The range of *R_Amstad_* is 0 (very difficult) to 100 (very easy).

As a second readability index the Erste Wiener Sachtextformel (*R_1. WSTF_*) was employed [19], which is based on the equation.

*R_1. WSTF_ = 0.1935 x MS + 0.1672 x SL + 0.1297 x lW - 0.0327 x OS - 0.875* where MS is the percentage of words with 3 or more syllables, SL the median sentence length (number of words per sentence), IW the percentage of words with> 6 characters, and OS the percentage of words with only one syllable. A low level score of R_1. WSTF_ is 4 (easy) whereas high levels scores of R 1. WSTF are values>12 (very difficult).

For each drug, three text passages ranging between 90 and 120 words were randomly selected in the German language version of Wikipedia and in the textbooks. Text passages were edited to exclude citations, headings or references. *R_Amstad_* and *R_1. WSTF_* were calculated using the koRpus text analysis software (http://ripley.psycho.hhu.de/koRpus).

### Statistics

Data are presented as means ± SEM. To test the statistical difference between means of two (t-test) or more groups (one-way ANOVA with Bonferroni correction) GraphPad PRISM 5.0 (La Jolla, CA) software was used. P values <0.05 were considered statistically significant.

## Results

### Structure and presentation of drug information in Wikipedia vs. textbooks

Of the 100 curricular drugs analyzed (**Table S1**), the majority (n = 95) was present as distinct articles in the German language version of Wikipedia. Only five drugs (cefepim, goserelin, unfractionated heparin, insulin lispro and repaglinide) were not covered as individual articles, but presented within an overview article of the particular drug family. Of note, the English language version of Wikipedia was more comprehensive and contained distinct articles on all drugs analyzed except of unfractionated heparin. Wikipedia articles had a heterogeneous structure with a varying number of subheadings. Nevertheless, most articles contained the subsections “indication”, “mechanism of action”, “adverse effects”, “contraindications” and “pharmacokinetics”. In addition, some articles embodied additional sections e.g. on patents and clinical trials, physicochemical properties or historical information. In contrast, the presentation of drug information in textbooks was more uniform and drugs were typically not discussed individually but portrayed as parts of pertinent drug families.

### Accuracy and Completeness

**Figure 2:**
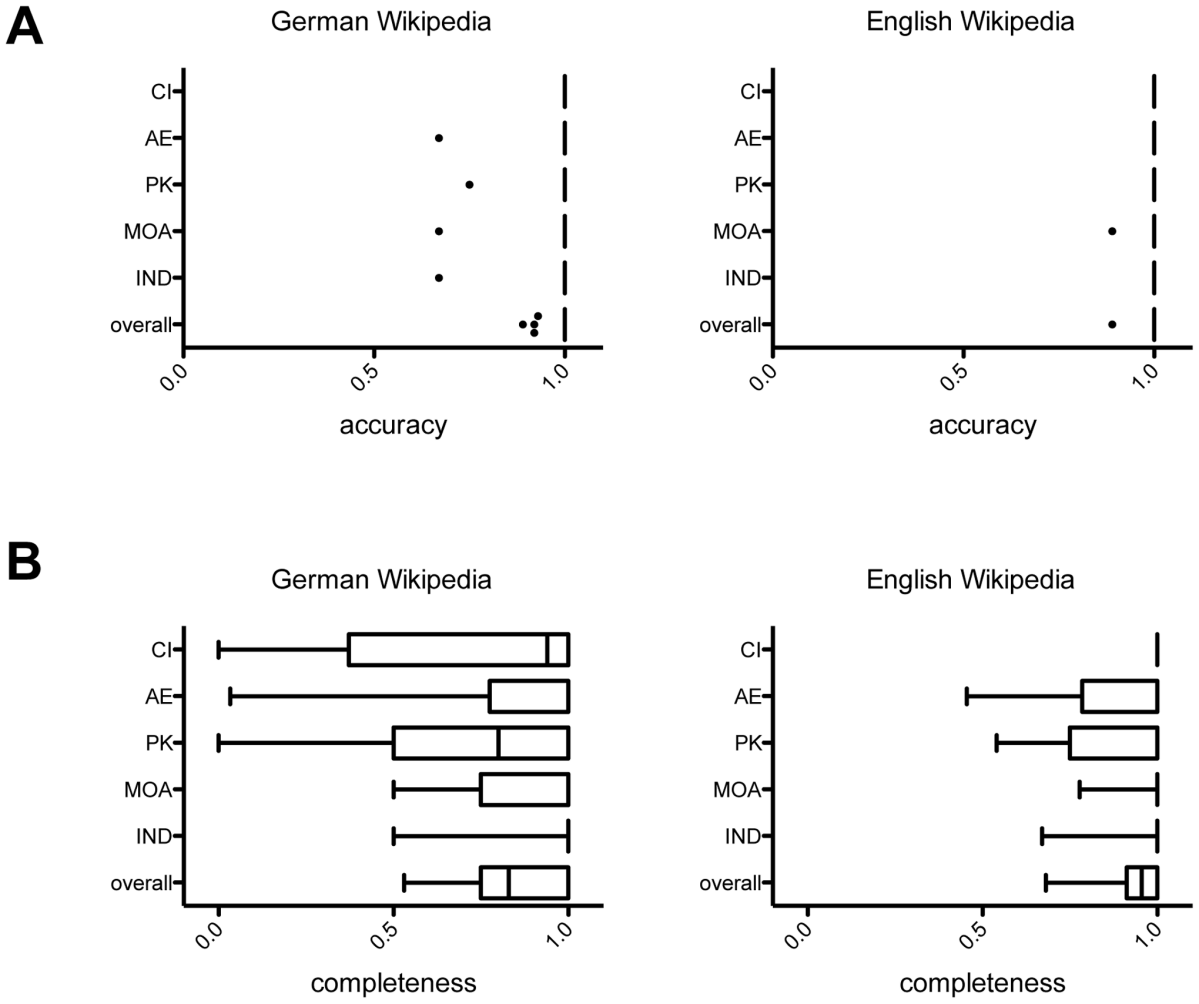
Accuracy and completeness of drug information in Wikipedia. Analysis of (A) accuracy and (B) completeness of the German (left panel) and English (right panel) language Wikipedia in comparison to textbooks. Accuracy was defined as the degree of closeness to the pharmacological information of the textbooks overlap. Completeness was defined as percentage of pharmacological statements of the textbook overlap present in the respective Wikipedia articles. Box plots showing median, first and third quartile with whiskers depicting the 5% and 95% percentile. In (A), statistical outliers are shown as black dots. Note that some box plots appear as vertical lines due to high accuracy scores and low variability of data. IND, indication; MA, mechanism of action; AE, adverse effects; PK, pharmacokinetics; CI, contraindications.

To investigate the accuracy of pharmacological information in Wikipedia, the percentage of correct pharmacological statements in Wikipedia was calculated in comparison to the textbook overlap (**Figure 1**). The textbook overlap was 55%±12% for the German textbooks and 74%±14% for the English textbooks. Accuracy of pharmacological information was in the range between 99.6 and 100% in the five categories evaluated for the German language version of Wikipedia, with an overall mean score of 99.7%±0.17% (**Figure 2A** **, Table S2**). Similar results were obtained with the English language version of Wikipedia (**Figure 2A** **, Table S2**).

Completeness of drug information in Wikipedia was quantified as percentage of the textbook overlap present in the respective Wikipedia drug articles. The overall completeness of drug information in the German language version of Wikipedia was 83.8%±1.5% (p<0.001) (**Figure 2B**) when compared to the textbook data overlap. Completeness varied in-between categories, and was lowest in the category “pharmacokinetics” (68.0%±4.2%; p<0.001) and highest in the category “indication” (91.3%±2.0%; ns) when compared to the textbook data overlap. Similar results were obtained for the English language version of Wikipedia with an overall completeness score of 93.1%±0.01% (**Figure 2B**). The most incomplete category was “pharmacokinetics” (87.2%±0.03%) when compared to the English textbook data overlap. It should be noted that the degree of overlap between the reference textbooks may affect the estimate of accuracy and completeness of drug information in Wikipedia. To minimize this effect, we used textbooks of comparable size and scope in the present study.

To assess if the information present in the textbook overlap but missing in Wikipedia (16.2% or a total of 224 pharmacological statements) was didactically relevant for undergraduate teaching of pharmacology, a qualitative re-evaluation was performed by six pharmacology lecturers of five different universities in Germany. Of the 224 statements evaluated, 84 (37.5%) were rated as relevant and 140 (62.5%) as non-relevant for a basic pharmacology course for medical students (**Table S3**). Thus, the adjusted overall completeness of pharmacological information for teaching in Wikipedia amounts to 93.9%.

Collectively, these results indicate that the drug information in Wikipedia is accurate and presents the majority (>90%) of curricular information essential for undergraduate medical education of pharmacology.

### References

**Figure 3:**
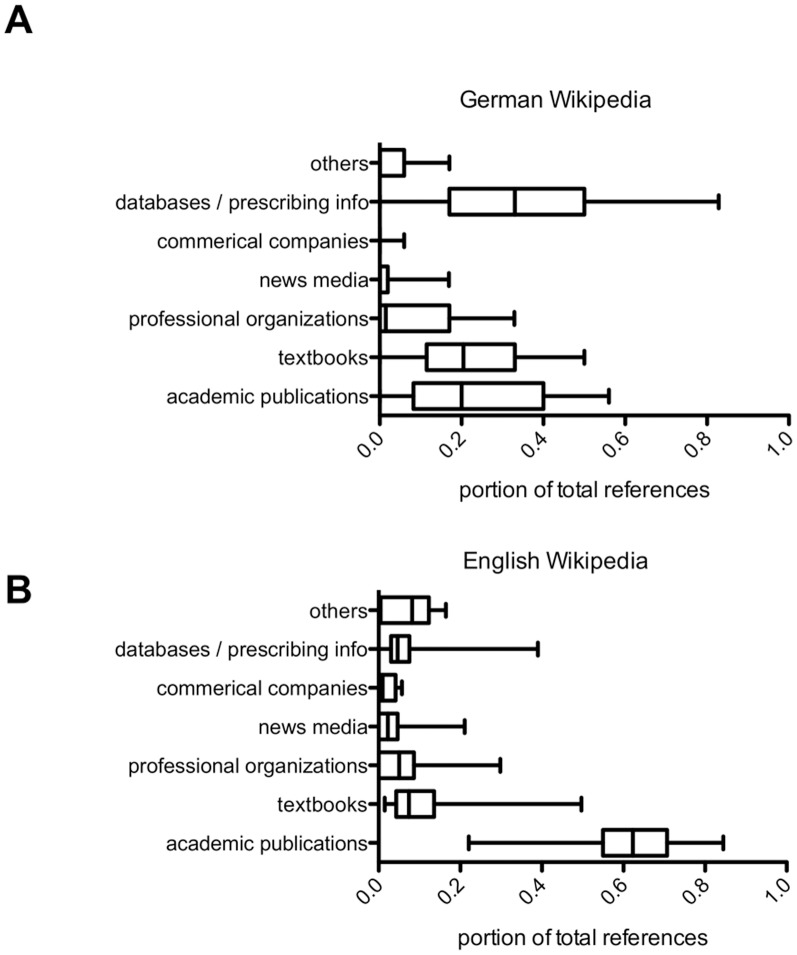
Analysis of article references in Wikipedia. (A) German language version of Wikipedia (n = 100), (B) English language version of Wikipedia (n = 20). Box plots depict the 5–95% percentile.

The average number of references per article in the German language version of Wikipedia was 14.6 (±1.6), with a minimum of 2 and the maximum of 104 references per article (**Table S4**). Most references were cited from reliable sources such as peer-reviewed academic publications (24.5%±0.02%), textbooks (22.9%±0.02%) or scientific databases and prescribing information (36.4%±0.02%). Non-peer reviewed or potentially biased information sources such as news media (online or print) or pharmaceutical industry accounted on average for only 3.2%±0.008% and 0.01%±0.005% of article references, respectively. **Figure 3** depicts the summative analysis of references of drug articles investigated. Similar results were obtained for references of the English language version of Wikipedia.

### Readability

**Figure 4:**
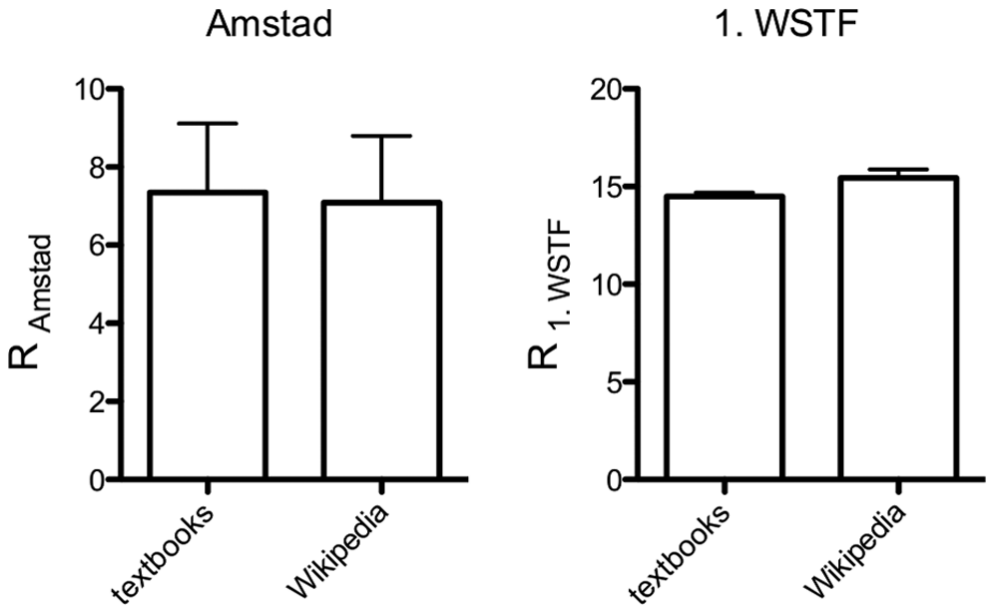
Analysis of readability. Readability was assessed using the Amstad readability index (*R_Amstad_*) and the Erste Wiener Sachtextformel (*R_1. WSTF_*). The scale of *R_Amstad_* is from 0 (very difficult) to 100 (easy to read), for *R_1. WSTF_* from 4 (easy to read) to >12 (very difficult). n = 10.

An important aspect for the comprehension of learning materials is their readability, which can be quantified by indices that assess sentence composition, complexity and length. Readability was assessed for Wikipedia and the textbooks using the Amstad readability index [18], a variant of the Flesch Reading Ease adapted for the evaluation of German texts, and the Wiener Sachtextformel 1 [19]. Overall, no significant difference in readability was found between Wikipedia and textbooks (*R_Amstad_*: 7.1±1.7 vs. 7.4±1.8, p = 0.9; *R_1. WSTF_*: 15.4±0.5 vs.14.5±0.2, p = 0.07; **Figure 4** **, Table S5**). Both media had readability scores indicative of difficult-to-read texts that require tertiary levels of education.

### Frequency of revisions of Wikipedia articles

In contrast to traditional encyclopedias or textbooks that are written and edited by professionals, Wikipedia allows any user to create and edit content. Drug articles in the German language version of Wikipedia had on average 262.8±37.4 edits performed by an average of 142.7±17.6 editors (**Table 1** **and S6**). The majority of editors were registered users of Wikipedia. In addition, Wikipedia articles were maintained by automated or semi-automated scripts (bot editors) that perform simple or structurally repetitive task e.g. removing outdated links or detecting vandalism. Bot edits accounted for approx. 7% of edits of the articles investigated. Of note, articles of general public interest (e.g. on the psychostimulant drug methylphenidate for the treatment of attention-deficit hyperactivity disorder) had significantly more editors and edits (637 and 1418, respectively).

**Table 1:** Descriptive statistics of edits and editors of drug articles in Wikipedia.

	Edits	Editors			
		All	Registered	Anonymous	Bots
**Minimum**	1	1	1	0	0
**Maximum**	2066	914	501	354	59
**Mean**	262.8	142.7	72.6	51.2	18.9
**SEM**	37.4	17.6	8.9	8.1	1.2
**Total**	24441	13266	6750	4757	1759

## Discussion

Since its launch in 2001 Wikipedia has become the largest collaborative online encyclopedia and is widely used by both students and educators in undergraduate medical education [4]. In this article we investigated the accuracy and completeness of drug information in Wikipedia in comparison to standard textbooks of basic pharmacology and evaluated its potential for undergraduate medical education of pharmacology. By analyzing 100 randomly selected curricular drugs in the German language version of Wikipedia, we found that the information in Wikipedia contained few factual errors (<0.3%) and comprised the majority (>93%) of curricular drug information considered essential for undergraduate teaching of basic pharmacology. The drug articles in Wikipedia had an average of 262.8±37.4 edits that were performed by an average of 142.7±17.6 editors. Finally, Wikipedia was generally well referenced with an average of 14.6±1.6 citations per article and had a readability that was on par with standard textbooks of pharmacology. Similar results were obtained for the English language version of Wikipedia. Collectively, our results suggest that Wikipedia is an accurate and informative source of drug information for undergraduate medical students.

As a collaborative encyclopedia with an open editorial policy, the reliability and accuracy of information of Wikipedia has frequently been the focus of intense debate (reviewed in [9]). An example is the single-blinded study by the journal *Nature* that compared the accuracy of 42 articles on various scientific topics in Wikipedia and *Encyclopedia Britannica* [20]. The authors identified a total of four severe errors in each of the encyclopedias. Subsequently, a number of empirical studies have assessed the quality of medical information in Wikipedia pertinent to surgical procedures [21], gastrointestinal conditions [22], cancer types [23] and pathology informatics [24]. Despite different methodologies, the main conclusion of these studies was that Wikipedia articles on health topics contain few errors and are well referenced, while the information provided often lacks depth.

Few studies have investigated the quality of pharmacological information in Wikipedia. Clauson et al. compared Wikipedia to the online database *Medscape drug reference* [10]. By using a set of predetermined questions, the authors found that Medscape provided answers to 82.5%, while Wikipedia could answer only 40%. In particular, Wikipedia articles were missing information on drug dosages, interactions and contraindications. However, it should be noted that the Wikipedia style manual for drug articles discourages detailed dosage information (http://en.wikipedia.org/wiki/Wikipedia:WikiProject_Pharmacology/Style_guide).

Kupferberg and co-worker analyzed the accuracy and completeness of pharmacological information of five different statin drugs [11]. The authors concluded that the drug information is less complete, inconsistent and cannot be recommended as sole information source. It is important to note that the aim of the studies by Clauson et al. and Kupferberg and Protus was to evaluate drug information for patients or health practitioners. In contrast, the present study aimed to analyze drug information in Wikipedia from an educators' perspective and therefore used standard textbooks of pharmacology as point of reference. Pharmacological information on drugs for educational purposes differs in focus and detail of pharmacodynamic and -kinetic content. Moreover, Wikipedia is evolving rapidly. Re-evaluating the drug entries assessed by Kupferberg and Protus in 2010, we found the quality of pharmacological information significantly improved (data not shown). Therefore, the major conclusion of the present study that Wikipedia is an accurate and informative source of drug information for undergraduate medical students, is not incompatible with previously reported findings.

The ease of updating and editing information is a potential strength of Wikipedia, in particular in rapidly evolving disciplines such as pharmacology. However, this may also bear the risk of errors, vandalism or manipulation. Wikipedia has implemented several safety measures and quality surveillance mechanisms to identify and repair spurious information. These include watch lists for recent changes by volunteer editors, page protection for articles that are likely to attract controversy or vandalism, or automated computer scripts (bots) that detect and repair copyright violations, simple vandalism, or spelling and grammatical errors (reviewed in [9].

The effectiveness of these quality surveillance measures has been evaluated in a study by Viegas and co-worker who analyzed the repair dynamics of substantial rewrites of vandalism in Wikipedia [25]. The authors showed that the majority of such acts were repaired within minutes by the Wikipedia community. Another study reported that 42% of damaged articles were repaired within one viewing, while 11% were still present after 100 viewings [26]. These results suggest that while the quality surveillance is remarkably effective, a residual risk remains. A middle way between a closed expert database and an open editorial system may be platforms such as TUM300 (http://www.tum300.de) that allow anonymous user feedback, yet require editorial approval of edits prior to publication.

There are several limitations to this study. In lack of a “golden standard” for the accuracy and comprehensiveness of pharmacological information for undergraduate medical education, we used well established pharmacology textbooks as the point of reference. However, these resources have not been formally evaluated for these criteria. In addition, we analyzed a limited number of drugs in the German and English language version of Wikipedia, and accuracy and completeness of pharmacological information may differ for other drugs. The present study is therefore exploratory in nature and serves as basis for future confirmatory studies with larger sample sizes. It is important to note that pharmacology training of undergraduate medical students is not limited to pharmacodynamic and –kinetic characteristics of drugs. It is not the intention of this study to evaluate the potential of Wikipedia to substitute other learning media, in particular textbooks. Finally, the results of this study cannot be directly extrapolated to other areas of pharmacology (e.g. clinical pharmacology) or to other medical disciplines.

Despite these limitations our results underscore that the collaborative and participatory design of Wikipedia does generate high quality information on pharmacology that is suitable for undergraduate medical education. From a didactic point of view, the possibility to actively participate in editing and contributing to information resources is likely to meet the expectations and preferences of today's student generation.

## Supporting Information

Table S1 **List of curricular drugs articles.**
(PDF)

Table S2 **Completeness and accuracy of drug information in Wikipedia.**
(PDF)

Table S3 **Qualitative evaluation of textbook information missing in the German Wikipedia.**
(PDF)

Table S4 **Proportion of references per drug article in Wikipedia.**
(PDF)

Table S5 **Readability scores for German Wikipedia.**
(PDF)

Table S6 **Number of edits and editors per drug article.**
(PDF)
