# Supplementary material for: Accuracy and Completeness of Drug Information in Wikipedia: A Comparison with Standard Textbooks of Pharmacology
Source: PLoS One. 2014 Sep 24;9(9):e106930. doi: 10.1371/journal.pone.0106930 (PMC4174509; doi:10.1371/journal.pone.0106930)
Supplement: Table S1 — List of curricular drugs articles. (PDF) [file pone.0106930.s001.pdf]

Table S1: List of curricular drugs articles.

Annotations: 1: drug article analyzed in the German language version of Wikipedia. 2: drug article analyzed in the English language version of Wikipedia. 3. No article available in the German language version of Wikipedia. 4. No article available in the English language version of Wikipedia. 5. drug presented only within a drug family article.

English	annotation
Acetylsalicylic acid	1, 2
Aciclovir	1, 2
Alendronic acid	1
Allopurinol	1, 2
Amantadine	1, 2
Amiodarone	1
Amitriptyline	1, 2
Amoxicillin	1, 2
Atropine	1, 2
Azathioprine	1, 2
Benzylpenicillin	1
Biperiden	1
Bromocriptine	1, 2
Buprenorphine	1, 2
Caffeine	1
Candesartan	1, 2
Carbamazepine	1, 2
Cefepime	1, 3, 5
Ciclosporin	1
Ciprofloxacin	1, 2
Clarithromycin	1
Clopidogrel	1, 2
Cocaine	1
Cyclophosphamide	1
Diazepam	1, 2
Digoxin	1
Domperidone	1, 2
Doxazosin	1, 2
Doxycycline	1
Enoxaparin sodium	1
Epinephrine	1
Estradiol	1
Ethambutol	1, 2
Ethanol	1
Exenatide	1
Finasteride	1, 2
Flucloxacillin	1
Fluconazole	1, 2
Flumazenil	1, 2
Furosemide	1
Gentamicin	1, 2
Glyceryl trinitrate	1
Goserelin	1, 3, 5
Heparin, unfractionated	1, 4, 5
Hydrochlorothiazide	1, 2
Ibuprofen	1, 2
Imipenem	1, 2
Insulin lispro	1, 3, 5
Isoflurane	1

Isoniazid	1, 2
Lamotrigine	1, 2
Levodopa	1
Lithium	1
Loperamide	1, 2
Metamizole	1
Metformin	1, 2
Methanol	1
Methotrexate	1, 2
Methyldopa	1
Methylphenidate	1
Metoclopramide	1, 2
Metoprolol	1, 2
Metronidazole	1, 2
Mifepristone	1
Mirtazapine	1, 2
Molsidomine	1
Morphine	1, 2
Naloxone	1
Nicotine	1
Nifedipine	1, 2
Norepinephrine	1
Omeprazole	1, 2
Ondansetron	1
Paclitaxel	1, 2
Pancuronium	1, 2
Perchlorate	1
Phenobarbital	1, 2
Physostigmine	1
Pilocarpine	1, 2
Piperacillin	1, 2
Prednisolone	1
Propofol	1, 2
Pyrazinamide	1
Ramipril	1, 2
Ranitidine	1, 2
Repaglinide	1, 3, 5
Rifampicin	1
Rituximab	1, 2
Rivaroxaban	1
Sitagliptin	1
Somatropin	1
Spironolactone	1, 2
Suxamethonium	1, 2
Tamoxifen	1
Tazobactam	1
Thiamazole (Methimazole)	1, 2
Tramadol	1
Valproic acid	1
Vancomycin	1, 2
Zidovudine	1
