# Supplementary material for: Accuracy and Completeness of Drug Information in Wikipedia: A Comparison with Standard Textbooks of Pharmacology
Source: PLoS One. 2014 Sep 24;9(9):e106930. doi: 10.1371/journal.pone.0106930 (PMC4174509; doi:10.1371/journal.pone.0106930)
Supplement: Table S2 — Completeness and accuracy of drug information in Wikipedia. (PDF) [file pone.0106930.s002.pdf]

**Table S2: Completeness and accuracy of drug information in Wikipedia.**

| German Wikipedia | Completeness | Accuracy |
|---|---|---|
| Acetylsalicylic acid | | |
| I | 75% | 100% |
| MA | 50% | 100% |
| PK | 100% | 100% |
| AE | 75% | 100% |
| CI | 100% | 100% |
| Total | 80% | 100% |
| Aciclovir | | |
| I | 100% | 100% |
| MA | 100% | 100% |
| PK | 100% | 100% |
| AE | 100% | 100% |
| CI | - | - |
| Total | 100% | 100% |
| Alendronic acid | | |
| I | 100% | 100% |
| MA | 75% | 100% |
| PK | 100% | 100% |
| AE | 100% | 100% |
| CI | - | - |
| Total | 94% | 100% |
| Allopurinol | | |
| I | 100% | 100% |
| MA | 75% | 100% |
| PK | 50% | 100% |
| AE | - | - |
| CI | - | - |
| Total | 75% | 100% |
| Amantadine | | |
| I | 100% | 67% |
| MA | 100% | 100% |
| PK | - | - |
| AE | 100% | 100% |
| CI | - | - |
| Total | 100% | 89% |
| Amiodarone | | |
| I | 100% | 100% |
| MA | 100% | 100% |
| PK | 100% | 100% |
| AE | 100% | 100% |
| CI | - | - |
| Total | 100% | 100% |
| Amitriptyline | | |
| I | 100% | 100% |
| MA | 100% | 100% |
| PK | 100% | 100% |
| AE | 100% | 100% |
| CI | - | - |
| Total | 100% | 100% |

| | | |
|---|---|---|
| Amoxicillin | | |
| I | 67% | 100% |
| MA | 100% | 100% |
| PK | 0% | - |
| AE | 100% | 100% |
| CI | - | - |
| Total | 67% | 100% |
| | | |
| Atropine | | |
| I | 80% | 100% |
| MA | 80% | 100% |
| PK | 67% | 100% |
| AE | 100% | 100% |
| CI | - | - |
| Total | 82% | 100% |
| | | |
| Azathioprine | | |
| I | 100% | 100% |
| MA | 100% | 100% |
| PK | 100% | 100% |
| AE | 100% | 100% |
| CI | - | - |
| Total | 100% | 100% |
| | | |
| Benzylpenicillin | | |
| I | 100% | 100% |
| MA | 100% | 100% |
| PK | 100% | 100% |
| AE | 67% | 100% |
| CI | - | - |
| Total | 92% | 100% |
| | | |
| Biperiden | | |
| I | 100% | 100% |
| MA | 50% | 100% |
| PK | - | - |
| AE | 100% | 100% |
| CI | - | - |
| Total | 83% | 100% |
| | | |
| Bromocriptine | | |
| I | 75% | 100% |
| MA | 100% | 100% |
| PK | - | - |
| AE | 80% | 100% |
| CI | - | - |
| Total | 85% | 100% |
| | | |
| Buprenorphine | | |
| I | 100% | 100% |
| MA | 100% | 100% |
| PK | 100% | 75% |
| AE | - | - |
| CI | - | - |
| Total | 100% | 92% |
| | | |
| Caffeine | | |
| I | 100% | 100% |
| MA | 90% | 100% |
| PK | 80% | 100% |
| AE | 43% | 100% |
| CI | - | - |
| Total | 78% | 100% |

| | | |
|---|---|---|
| Candesartan | | |
| I | 100% | 100% |
| MA | 100% | 100% |
| PK | 0% | - |
| AE | - | - |
| CI | - | - |
| Total | 67% | 100% |
| | | |
| Carbamazepine | | |
| I | 100% | 100% |
| MA | 50% | 100% |
| PK | 100% | 100% |
| AE | 100% | 100% |
| CI | - | - |
| Total | 88% | 100% |
| | | |
| Cefepim | | |
| I | 0% | - |
| MA | 100% | 100% |
| PK | 100% | 100% |
| AE | 100% | 100% |
| CI | - | - |
| Total | 75% | 100% |
| | | |
| Ciclosporin | | |
| I | 50% | 100% |
| MA | 100% | 100% |
| PK | 0% | - |
| AE | 83% | 100% |
| CI | - | - |
| Total | 58% | 100% |
| | | |
| Ciprofloxacin | | |
| I | 100% | 100% |
| MA | 67% | 100% |
| PK | 50% | 100% |
| AE | 100% | 100% |
| CI | 67% | 100% |
| Total | 77% | 100% |
| | | |
| Clarithromycin | | |
| I | 80% | 100% |
| MA | 100% | 100% |
| PK | 100% | 100% |
| AE | 100% | 100% |
| CI | - | - |
| Total | 95% | 100% |
| | | |
| Clopidogrel | | |
| I | 100% | 100% |
| MA | 100% | 100% |
| PK | 100% | 100% |
| AE | 100% | 100% |
| CI | - | - |
| Total | 100% | 100% |
| | | |
| Cocaine | | |
| I | - | - |
| MA | 100% | 100% |
| PK | 100% | 100% |
| AE | 100% | 100% |
| CI | - | - |
| Total | 100% | 100% |

| | | |
|---|---|---|
| Cyclophophamide | | |
| I | 100% | 100% |
| MA | 67% | 100% |
| PK | 100% | 100% |
| AE | 100% | 100% |
| CI | - | - |
| Total | 92% | 100% |
| Diazepam | | |
| I | 100% | 100% |
| MA | 50% | 100% |
| PK | 50% | 100% |
| AE | 71% | 100% |
| CI | 100% | 100% |
| Total | 74% | 100% |
| Digoxin | | |
| I | 100% | 100% |
| MA | 75% | 100% |
| PK | 75% | 100% |
| AE | 100% | 100% |
| CI | 0% | - |
| Total | 70% | 100% |
| Domperidone | | |
| I | 33% | 100% |
| MA | 100% | 100% |
| PK | 100% | 100% |
| AE | - | - |
| CI | - | - |
| Total | 78% | 100% |
| Doxazosin | | |
| I | 100% | 100% |
| MA | 100% | 100% |
| PK | - | - |
| AE | 33% | 100% |
| CI | - | - |
| Total | 78% | 100% |
| Doxycycline | | |
| I | 56% | 100% |
| MA | 100% | 100% |
| PK | 57% | 100% |
| AE | 50% | 100% |
| CI | 100% | 100% |
| Total | 73% | 100% |
| Enoxaparin sodium | | |
| I | 100% | 100% |
| MA | 100% | 100% |
| PK | 100% | 100% |
| AE | 100% | 100% |
| CI | - | - |
| Total | 100% | 100% |
| Epinephrine | | |
| I | 100% | 100% |
| MA | 100% | 100% |
| PK | 100% | 100% |
| AE | 100% | 100% |
| CI | - | - |
| Total | 100% | 100% |

| | | |
|---|---|---|
| Estradiol | | |
| I | 50% | 100% |
| MA | 60% | 100% |
| PK | 0% | - |
| AE | 100% | 100% |
| CI | - | - |
| Total | 53% | 100% |
| | | |
| Ethambutol | | |
| I | 100% | 100% |
| MA | 100% | 100% |
| PK | 100% | 100% |
| AE | 100% | 100% |
| CI | - | - |
| Total | 100% | 100% |
| | | |
| Ethanol | | |
| I | - | - |
| MA | 50% | 100% |
| PK | 56% | 100% |
| AE | 100% | 100% |
| CI | - | - |
| Total | 69% | 100% |
| | | |
| Exenatide | | |
| I | 100% | 100% |
| MA | 100% | 100% |
| PK | - | - |
| AE | 100% | 100% |
| CI | - | - |
| Total | 100% | 100% |
| | | |
| Finasteride | | |
| I | 100% | 100% |
| MA | 100% | 100% |
| PK | - | - |
| AE | - | - |
| CI | - | - |
| Total | 100% | 100% |
| | | |
| Flucloxacillin | | |
| I | 100% | 100% |
| MA | 100% | 100% |
| PK | 20% | 100% |
| AE | - | - |
| CI | - | - |
| Total | 73% | 100% |
| | | |
| Fluconazole | | |
| I | 100% | 100% |
| MA | 100% | 100% |
| PK | 0% | - |
| AE | 100% | 100% |
| CI | - | - |
| Total | 75% | 100% |
| | | |
| Flumazenil | | |
| I | 100% | 100% |
| MA | 100% | 100% |
| PK | 50% | 100% |
| AE | - | - |
| CI | - | - |
| Total | 83% | 100% |

| | | |
|---|---|---|
| Furosemide | | |
| I | 100% | 100% |
| MA | 100% | 100% |
| PK | 75% | 100% |
| AE | 80% | 100% |
| CI | - | - |
| Total | 89% | 100% |
| | | |
| Gentamicin | | |
| I | 100% | 100% |
| MA | 67% | 100% |
| PK | 29% | 100% |
| AE | 100% | 100% |
| CI | 50% | 100% |
| Total | 69% | 100% |
| | | |
| Glyceryl trinitrate | | |
| I | 100% | 100% |
| MA | 100% | 100% |
| PK | 75% | 100% |
| AE | 100% | 100% |
| CI | - | - |
| Total | 94% | 100% |
| | | |
| Goserelin | | |
| I | 100% | 100% |
| MA | 50% | 100% |
| PK | - | - |
| AE | 0% | - |
| CI | - | - |
| Total | 50% | 100% |
| | | |
| Heparin, unfractionated | | |
| I | 100% | 100% |
| MA | 100% | 100% |
| PK | 100% | 100% |
| AE | 50% | 100% |
| CI | 100% | 100% |
| Total | 90% | 100% |
| | | |
| Hydrochlorothiazide | | |
| I | 67% | 100% |
| MA | 100% | 100% |
| PK | 50% | 100% |
| AE | 100% | 100% |
| CI | - | - |
| Total | 79% | 100% |
| | | |
| Ibuprofen | | |
| I | 100% | 100% |
| MA | 100% | 100% |
| PK | 0% | - |
| AE | 100% | 100% |
| CI | - | - |
| Total | 75% | 100% |
| | | |
| Imipenem | | |
| I | 100% | 100% |
| MA | 100% | 100% |
| PK | 80% | 100% |
| AE | 0% | - |
| CI | - | - |
| Total | 70% | 100% |

| | | |
|---|---|---|
| Insulin lispro | | |
| I | 100% | 100% |
| MA | 100% | 100% |
| PK | 33% | 100% |
| AE | 100% | 100% |
| CI | - | - |
| Total | 83% | 100% |
| | | |
| Isoflurane | | |
| I | 100% | 100% |
| MA | 100% | 100% |
| PK | 25% | 100% |
| AE | - | - |
| CI | - | - |
| Total | 75% | 100% |
| | | |
| Isoniazid | | |
| I | 100% | 100% |
| MA | 100% | 100% |
| PK | 75% | 100% |
| AE | 100% | 100% |
| CI | - | - |
| Total | 94% | 100% |
| | | |
| Lamotrigine | | |
| I | 33% | 100% |
| MA | 100% | 100% |
| PK | - | - |
| AE | 100% | 100% |
| CI | - | - |
| Total | 78% | 100% |
| | | |
| Levodopa | | |
| I | 100% | 100% |
| MA | 67% | 100% |
| PK | - | - |
| AE | 50% | 100% |
| CI | - | - |
| Total | 72% | 100% |
| | | |
| Lithium | | |
| I | 75% | 100% |
| MA | 100% | 100% |
| PK | 50% | 100% |
| AE | 50% | 100% |
| CI | 100% | 100% |
| Total | 75% | 100% |
| | | |
| Loperamide | | |
| I | 100% | 100% |
| MA | 0% | - |
| PK | 100% | 100% |
| AE | - | - |
| CI | 0% | - |
| Total | 50% | 100% |
| | | |
| Metamizole | | |
| I | 100% | 100% |
| MA | 100% | 100% |
| PK | 100% | 100% |
| AE | 100% | 100% |
| CI | - | - |
| Total | 100% | 100% |

| | | |
|---|---|---|
| Metformin | | |
| I | 100% | 100% |
| MA | 100% | 100% |
| PK | 0% | - |
| AE | 100% | 100% |
| CI | 88% | 100% |
| Total | 78% | 100% |
| | | |
| Methanol | | |
| I | - | - |
| MA | 100% | 100% |
| PK | 100% | 100% |
| AE | - | - |
| CI | - | - |
| Total | 100% | 100% |
| | | |
| Methotrexate | | |
| I | 100% | 100% |
| MA | 75% | 100% |
| PK | 100% | 100% |
| AE | 80% | 100% |
| CI | - | - |
| Total | 89% | 100% |
| | | |
| Methyldopa | | |
| I | 100% | 100% |
| MA | 100% | 100% |
| PK | 100% | 100% |
| AE | 100% | 100% |
| CI | - | - |
| Total | 100% | 100% |
| | | |
| Methylphenidate | | |
| I | 100% | 100% |
| MA | 100% | 100% |
| PK | - | - |
| AE | 100% | 100% |
| CI | - | - |
| Total | 100% | 100% |
| | | |
| Metoclopramide | | |
| I | 100% | 100% |
| MA | 67% | 100% |
| PK | - | - |
| AE | 50% | 100% |
| CI | - | - |
| Total | 72% | 100% |
| | | |
| Metoprolol | | |
| I | 100% | 100% |
| MA | 100% | 100% |
| PK | 67% | 100% |
| AE | 100% | 67% |
| CI | 100% | 100% |
| Total | 93% | 93% |
| | | |
| Metronidazole | | |
| I | 67% | 100% |
| MA | 100% | 100% |
| PK | 100% | 100% |
| AE | 100% | 100% |
| CI | 100% | 100% |
| Total | 93% | 100% |

| | | |
|---|---|---|
| Mifepristone | | |
| I | 100% | 100% |
| MA | 100% | 100% |
| PK | - | - |
| AE | - | - |
| CI | - | - |
| Total | 100% | 100% |
| | | |
| Mirtazapine | | |
| I | 50% | 100% |
| MA | 100% | 100% |
| PK | - | - |
| AE | - | - |
| CI | - | - |
| Total | 75% | 100% |
| | | |
| Molsidomine | | |
| I | 100% | 100% |
| MA | 100% | 67% |
| PK | 100% | 100% |
| AE | 100% | 100% |
| CI | - | - |
| Total | 100% | 92% |
| | | |
| Morphine | | |
| I | 100% | 100% |
| MA | 80% | 100% |
| PK | 25% | 100% |
| AE | 50% | 100% |
| CI | 0% | - |
| Total | 51% | 100% |
| | | |
| Naloxone | | |
| I | 50% | 100% |
| MA | 100% | 100% |
| PK | 50% | 100% |
| AE | 100% | 100% |
| CI | - | - |
| Total | 75% | 100% |
| | | |
| Nicotine | | |
| I | - | - |
| MA | 80% | 100% |
| PK | 50% | 100% |
| AE | 33% | 100% |
| CI | - | - |
| Total | 54% | 100% |
| | | |
| Nifedipine | | |
| I | 100% | 100% |
| MA | 100% | 100% |
| PK | 0% | - |
| AE | 100% | 100% |
| CI | - | - |
| Total | 75% | 100% |
| | | |
| Norepinephrine | | |
| I | 100% | 100% |
| MA | 50% | 100% |
| PK | 67% | 100% |
| AE | 100% | 100% |
| CI | - | - |
| Total | 79% | 100% |

| | | |
|---|---|---|
| Omeprazole | | |
| I | 100% | 100% |
| MA | 50% | 100% |
| PK | 80% | 100% |
| AE | 80% | 100% |
| CI | - | - |
| Total | 78% | 100% |
| | | |
| Ondansetron | | |
| I | 100% | 100% |
| MA | 100% | 100% |
| PK | 25% | 100% |
| AE | 100% | 100% |
| CI | - | - |
| Total | 81% | 100% |
| | | |
| Paclitaxel | | |
| I | 100% | 100% |
| MA | 100% | 100% |
| PK | - | - |
| AE | 100% | 100% |
| CI | - | - |
| Total | 100% | 100% |
| | | |
| Pancuronium | | |
| I | 50% | 100% |
| MA | 100% | 100% |
| PK | 100% | 100% |
| AE | 100% | 100% |
| CI | - | - |
| Total | 88% | 100% |
| | | |
| Perchlorate | | |
| I | 100% | 100% |
| MA | 100% | 100% |
| PK | - | - |
| AE | 0% | - |
| CI | - | - |
| Total | 67% | 100% |
| | | |
| Phenobarbital | | |
| I | 100% | 100% |
| MA | 100% | 100% |
| PK | 100% | 100% |
| AE | 100% | 100% |
| CI | - | - |
| Total | 100% | 100% |
| | | |
| Physostigmine | | |
| I | 100% | 100% |
| MA | 75% | 100% |
| PK | 100% | 100% |
| AE | 100% | 100% |
| CI | - | - |
| Total | 94% | 100% |
| | | |
| Pilocarpine | | |
| I | 100% | 100% |
| MA | 67% | 100% |
| PK | 100% | 100% |
| AE | 0% | - |
| CI | - | - |
| Total | 67% | 100% |

| | | |
|---|---|---|
| Piperacillin | | |
| I | 100% | 100% |
| MA | 100% | 100% |
| PK | 100% | 100% |
| AE | - | - |
| CI | - | - |
| Total | 100% | 100% |
| | | |
| Prednisolone | | |
| I | 86% | 100% |
| MA | 43% | 100% |
| PK | 0% | - |
| AE | 45% | 100% |
| CI | 50% | 100% |
| Total | 45% | 100% |
| | | |
| Propofol | | |
| I | 100% | 100% |
| MA | 100% | 100% |
| PK | 0% | - |
| AE | 100% | 100% |
| CI | - | - |
| Total | 75% | 100% |
| | | |
| Pyrazinamide | | |
| I | 100% | 100% |
| MA | 33% | 100% |
| PK | 75% | 100% |
| AE | 100% | 100% |
| CI | - | - |
| Total | 77% | 100% |
| | | |
| Ramipril | | |
| I | 100% | 100% |
| MA | 100% | 100% |
| PK | - | - |
| AE | 100% | 100% |
| CI | 100% | 100% |
| Total | 100% | 100% |
| | | |
| Ranitidine | | |
| I | 100% | 100% |
| MA | 100% | 100% |
| PK | - | - |
| AE | 67% | 100% |
| CI | - | - |
| Total | 89% | 100% |
| | | |
| Repaglinide | | |
| I | 100% | 100% |
| MA | 100% | 100% |
| PK | 100% | 100% |
| AE | 100% | 100% |
| CI | - | - |
| Total | 100% | 100% |
| | | |
| Rifampicin | | |
| I | 100% | 100% |
| MA | 100% | 100% |
| PK | 0% | - |
| AE | 43% | 100% |
| CI | 33% | 100% |
| Total | 55% | 100% |

| | | |
|---|---|---|
| Rituximab | | |
| I | 100% | 100% |
| MA | 100% | 100% |
| PK | - | - |
| AE | - | - |
| CI | - | - |
| Total | 100% | 100% |
| Rivaroxaban | | |
| I | 100% | 100% |
| MA | 100% | 100% |
| PK | 100% | 100% |
| AE | - | - |
| CI | - | - |
| Total | 100% | 100% |
| Sitagliptin | | |
| I | 100% | 100% |
| MA | 100% | 100% |
| PK | 100% | 100% |
| AE | - | - |
| CI | - | - |
| Total | 100% | 100% |
| Somatotropin | | |
| I | 100% | 100% |
| MA | 100% | 100% |
| PK | - | - |
| AE | - | - |
| CI | - | - |
| Total | 100% | 100% |
| Spironolactone | | |
| I | 100% | 100% |
| MA | 100% | 100% |
| PK | 100% | 100% |
| AE | 100% | 100% |
| CI | - | - |
| Total | 100% | 100% |
| Suxamethonium | | |
| I | 100% | 100% |
| MA | 75% | 100% |
| PK | 100% | 100% |
| AE | 80% | 100% |
| CI | - | - |
| Total | 89% | 100% |
| Tamoxifen | | |
| I | 100% | 100% |
| MA | 100% | 100% |
| PK | - | - |
| AE | 100% | 100% |
| CI | - | - |
| Total | 100% | 100% |
| Tazobactam | | |
| I | 50% | 100% |
| MA | 100% | 100% |
| PK | 100% | 100% |
| AE | - | - |
| CI | - | - |
| Total | 83% | 100% |

| | | |
|---|---|---|
| Thiamazole (Methimazole) | | |
| I | 100% | 100% |
| MA | 50% | 100% |
| PK | 0% | - |
| AE | 100% | 100% |
| CI | - | - |
| Total | 63% | 100% |
| | | |
| Tramadol | | |
| I | 100% | 100% |
| MA | 100% | 100% |
| PK | 100% | 100% |
| AE | - | - |
| CI | - | - |
| Total | 100% | 100% |
| | | |
| Valproic acid | | |
| I | 67% | 100% |
| MA | 50% | 100% |
| PK | 50% | 100% |
| AE | 100% | 100% |
| CI | - | - |
| Total | 67% | 100% |
| | | |
| Vancomycin | | |
| I | 100% | 100% |
| MA | 100% | 100% |
| PK | 33% | 100% |
| AE | 67% | 100% |
| CI | - | - |
| Total | 75% | 100% |
| | | |
| Zidovudine | | |
| I | 100% | 100% |
| MA | 100% | 100% |
| PK | - | - |
| AE | 100% | 100% |
| CI | - | - |
| Total | 100% | 100% |

| **English Wikipedia** | **Completeness** | **Accuracy** |
|---|---|---|
| Acetylsalicylic acid | | |
| I | 100% | 100% |
| MOA | 100% | 100% |
| PK | 75% | 100% |
| AE | 44% | 100% |
| CI | - | - |
| Total | 80% | 100% |
| | | |
| Aciclovir | | |
| I | 100% | 100% |
| MOA | 100% | 100% |
| PK | 83% | 100% |
| AE | 100% | 100% |
| CI | - | - |

| | | |
|---|---|---|
| Total | 96% | 100% |
| | | |
| Allopurinol | | |
| I | 100% | 100% |
| MOA | 100% | 100% |
| PK | 75% | 100% |
| AE | 100% | 100% |
| CI | - | - |
| Total | 94% | 100% |
| | | |
| Amantadine | | |
| I | 50% | 100% |
| MOA | 100% | 100% |
| PK | - | - |
| AE | 50% | 100% |
| CI | - | - |
| Total | 67% | 100% |
| | | |
| Amitriptyline | | |
| I | 100% | 100% |
| MOA | 100% | 100% |
| PK | 80% | 100% |
| AE | 100% | 100% |
| CI | - | - |
| Total | 95% | 100% |
| | | |
| Amoxicillin | | |
| I | 100% | 100% |
| MOA | 100% | 100% |
| PK | 0% | |
| AE | 83% | 100% |
| CI | - | - |
| Total | 71% | 100% |
| | | |
| Atropine | | |
| I | 100% | 100% |
| MOA | 80% | 100% |
| PK | 100% | 100% |
| AE | 100% | 100% |
| CI | 100% | 100% |
| Total | 96% | 100% |
| | | |
| Azathioprine | | |
| I | 100% | 100% |
| MOA | 100% | 100% |
| PK | 100% | 100% |
| AE | 75% | 100% |
| CI | - | - |
| Total | 94% | 100% |
| | | |
| Bromocriptine | | |
| I | 100% | 100% |
| MOA | 100% | 67% |
| PK | - | - |
| AE | 100% | 100% |
| CI | - | - |
| Total | 100% | 89% |
| | | |
| Buprenorphine | | |
| I | 100% | 100% |
| MOA | 100% | 100% |
| PK | 100% | 100% |
| AE | - | - |
| CI | - | - |

| | | |
|---|---|---|
| Total | 100% | 100% |
| | | |
| Candesartan | | |
| I | 100% | 100% |
| MOA | 100% | 100% |
| PK | - | - |
| AE | 100% | 100% |
| CI | - | - |
| Total | 100% | 100% |
| | | |
| Carpamazepine | | |
| I | 100% | 100% |
| MOA | 100% | 100% |
| PK | 100% | 100% |
| AE | 67% | 100% |
| CI | - | - |
| Total | 92% | 100% |
| | | |
| Ciprofloxacin | | |
| I | 71% | 100% |
| MOA | 100% | 100% |
| PK | 100% | 100% |
| AE | 100% | 100% |
| CI | - | - |
| Total | 93% | 100% |
| | | |
| Clopidogrel | | |
| I | 100% | 100% |
| MOA | 100% | 100% |
| PK | - | - |
| AE | 100% | 100% |
| CI | - | - |
| Total | 100% | 100% |
| | | |
| Diazepam | | |
| I | 100% | 100% |
| MOA | 100% | 100% |
| PK | 100% | 100% |
| AE | 50% | 100% |
| CI | - | - |
| Total | 88% | 100% |
| | | |
| Domperidone | | |
| I | 100% | 100% |
| MOA | 100% | 100% |
| PK | 100% | 100% |
| AE | - | - |
| CI | - | - |
| Total | 100% | 100% |
| | | |
| Doxazosin | | |
| I | 100% | 100% |
| MOA | 75% | 100% |
| PK | 100% | 100% |
| AE | 0% | - |
| CI | - | - |
| Total | 69% | 100% |
| | | |
| Ethambutol | | |
| I | 100% | 100% |
| MOA | 100% | 100% |
| PK | 67% | 100% |
| AE | 100% | 100% |
| CI | - | - |

| | | |
|---|---|---|
| Total | 92% | 100% |
| | | |
| Finasteride | | |
| I | 100% | 100% |
| MOA | 100% | 100% |
| PK | 100% | 100% |
| AE | - | - |
| CI | - | - |
| Total | 100% | 100% |
| | | |
| Fluconazole | | |
| I | 100% | 100% |
| MOA | 100% | 100% |
| PK | 75% | 100% |
| AE | 100% | 100% |
| CI | - | - |
| Total | 94% | 100% |
| | | |
| Flumazenil | | |
| I | 100% | 100% |
| MOA | 100% | 100% |
| PK | - | - |
| AE | 100% | 100% |
| CI | - | - |
| Total | 100% | 100% |
| | | |
| Gentamicin | | |
| I | 67% | 100% |
| MOA | 100% | 100% |
| PK | 60% | 100% |
| AE | 100% | 100% |
| CI | - | - |
| Total | 82% | 100% |
| | | |
| Hydrochlorthiazide | | |
| I | 100% | 100% |
| MOA | 100% | 100% |
| PK | 67% | 100% |
| AE | 67% | 100% |
| CI | - | - |
| Total | 83% | 100% |
| | | |
| Ibuprofen | | |
| I | 67% | 100% |
| MOA | 100% | 100% |
| PK | - | - |
| AE | 90% | 100% |
| CI | - | - |
| Total | 86% | 100% |
| | | |
| Imipenem | | |
| I | 100% | 100% |
| MOA | 100% | 100% |
| PK | 100% | 100% |
| AE | 100% | 100% |
| CI | - | - |
| Total | 100% | 100% |
| | | |
| Isoniazid | | |
| I | 100% | 100% |
| MOA | 100% | 100% |
| PK | 80% | 100% |
| AE | 75% | 100% |
| CI | - | - |

| | | |
|---|---|---|
| Total | 89% | 100% |
| | | |
| Lamotrigine | | |
| I | 100% | 100% |
| MOA | 100% | 100% |
| PK | 100% | 100% |
| AE | 100% | 100% |
| CI | - | - |
| Total | 100% | 100% |
| | | |
| Loperamide | | |
| I | 100% | 100% |
| MOA | 100% | 100% |
| PK | - | - |
| AE | - | - |
| CI | - | - |
| Total | 100% | 100% |
| | | |
| Metformin | | |
| I | 100% | 100% |
| MOA | 100% | 100% |
| PK | 100% | 100% |
| AE | 100% | 100% |
| CI | 100% | 100% |
| Total | 100% | 100% |
| | | |
| Methorexate | | |
| I | 80% | 100% |
| MOA | 100% | 100% |
| PK | 80% | 100% |
| AE | 80% | 100% |
| CI | 100% | 100% |
| Total | 85% | 100% |
| | | |
| Metoclopramide | | |
| I | 100% | 100% |
| MOA | 100% | 100% |
| PK | 100% | 100% |
| AE | 67% | 100% |
| CI | - | - |
| Total | 92% | 100% |
| | | |
| Metoprolol | | |
| I | 100% | 100% |
| MOA | 100% | 100% |
| PK | 67% | 100% |
| AE | 100% | 100% |
| CI | - | - |
| Total | 92% | 100% |
| | | |
| Metronidazole | | |
| I | 100% | 100% |
| MOA | 100% | 100% |
| PK | 67% | 100% |
| AE | 100% | 100% |
| CI | - | - |
| Total | 92% | 100% |
| | | |
| Mirtazapine | | |
| I | 100% | 100% |
| MOA | 100% | 100% |
| PK | 100% | 100% |
| AE | 100% | 100% |

| | | |
|---|---|---|
| CI | - | - |
| Total | 100% | 100% |
| | | |
| Morphine | | |
| I | 100% | 100% |
| MOA | 100% | 100% |
| PK | 100% | 100% |
| AE | 78% | 100% |
| CI | 100% | 100% |
| Total | 94% | 100% |
| | | |
| Nifedipine | | |
| I | 100% | 100% |
| MOA | 25% | 100% |
| PK | - | - |
| AE | - | - |
| CI | - | - |
| Total | 63% | 100% |
| | | |
| Omeprazole | | |
| I | 100% | 100% |
| MOA | 100% | 100% |
| PK | 75% | 100% |
| AE | 100% | 100% |
| CI | - | - |
| Total | 94% | 100% |
| | | |
| Paclitaxel | | |
| I | 100% | 100% |
| MOA | 100% | 100% |
| PK | - | - |
| AE | 100% | 100% |
| CI | - | - |
| Total | 100% | 100% |
| | | |
| Pancuronium | | |
| I | 100% | 100% |
| MOA | 100% | 100% |
| PK | 100% | 100% |
| AE | 100% | 100% |
| CI | - | - |
| Total | 100% | 100% |
| | | |
| Phenobarbital | | |
| I | 100% | 100% |
| MOA | 100% | 100% |
| PK | 100% | 100% |
| AE | 100% | 100% |
| CI | 100% | 100% |
| Total | 100% | 100% |
| | | |
| Pilocarpine | | |
| I | 100% | 100% |
| MOA | 100% | 100% |
| PK | - | - |
| AE | - | - |
| CI | - | - |
| Total | 100% | 100% |
| | | |
| Piperacillin | | |
| I | 100% | 100% |
| MOA | 100% | 100% |
| PK | - | - |
| AE | 100% | 100% |

| | | |
|---|---|---|
| CI | - | - |
| Total | 100% | 100% |
| | | |
| Propofol | | |
| I | 100% | 100% |
| MOA | 100% | 100% |
| PK | 100% | 100% |
| AE | 100% | 100% |
| CI | - | - |
| Total | 100% | 100% |
| | | |
| Ramipril | | |
| I | 100% | 100% |
| MOA | 100% | 100% |
| PK | - | - |
| AE | 100% | 100% |
| CI | - | - |
| Total | 100% | 100% |
| | | |
| Ranitidine | | |
| I | 100% | 100% |
| MOA | 100% | 100% |
| PK | 100% | 100% |
| AE | 67% | 100% |
| CI | - | - |
| Total | 92% | 100% |
| | | |
| Rituximab | | |
| I | 100% | 100% |
| MOA | 100% | 100% |
| PK | - | - |
| AE | 100% | 100% |
| CI | - | - |
| Total | 100% | 100% |
| | | |
| Spironolactone | | |
| I | 100% | 100% |
| MOA | 100% | 100% |
| PK | 100% | 100% |
| AE | 100% | 100% |
| CI | - | - |
| Total | 100% | 100% |
| | | |
| Suxamethonium | | |
| I | 100% | 100% |
| MOA | 100% | 100% |
| PK | 100% | 100% |
| AE | 100% | 100% |
| CI | - | - |
| Total | 100% | 100% |
| | | |
| Thiamazole (Methimazole) | | |
| I | 100% | 100% |
| MOA | 100% | 100% |
| PK | 75% | 100% |
| AE | 80% | 100% |
| CI | - | - |
| Total | 89% | 100% |
| | | |
| Vancomycin | | |
| I | 100% | 100% |
| MOA | 100% | 100% |
| PK | 100% | 100% |
| AE | 100% | 100% |

| | | |
|---|---|---|
| CI | - | - |
| Total | 100% | 100% |
