# Supplementary material for: Accuracy and Completeness of Drug Information in Wikipedia: A Comparison with Standard Textbooks of Pharmacology
Source: PLoS One. 2014 Sep 24;9(9):e106930. doi: 10.1371/journal.pone.0106930 (PMC4174509; doi:10.1371/journal.pone.0106930)
Supplement: Table S3 — Qualitative evaluation of textbook information missing in the German Wikipedia. (PDF) [file pone.0106930.s003.pdf]

Table S3: Qualitative evaluation of textbook information missing in the German Wikipedia.

Textbook information not present in Wikipedia (keywords)	Evaluation by lecturers (A-F) (1= didactically relevant; 0 = didactically not relevant)							didactic relevance (yes if sum > 4)
	A	B	C	D	E	F	sum	result
**Acetylsalicylic acid**								
Acute coronary syndrome	1	1	1	1	1	1	**6**	yes
Migraine attack	1	1	1	0	1	0	**4**	no
Inhibition of endothelial prostacyclin synthesis	1	1	0	0	1	1	**4**	no
CNS side effects (high doses)	0	1	0	0	1	0	**2**	no
**Alendronic acid**								
Osteoclast uptake	1	1	1	1	0	1	**5**	yes
**Allopurinol**								
Isomere of hypoxanthin	1	0	1	0	0	1	**3**	no
Oral application	0	1	0	0	1	1	**3**	no
**Amoxicillin**								
H. influenzae infections	0	1	1	1	0	1	**4**	no
High oral resoption when compared to Ampicillin	1	0	1	1	1	1	**5**	yes
**Atropine**								
Iridolysis	0	0	0	0	1	1	**2**	no
Mydriasis	1	1	0	1	1	1	**5**	yes
50% renal elimination	0	0	0	0	0	0	**0**	no
**Benzylpenicillin**								
Neurotoxity (high doses)	1	1	0	0	1	0	**3**	no
**Biperiden**								
IND: M. Parkinson	1	1	1	1	1	1	**6**	yes
**Bromocriptine**								
Increased GH incretion	1	0	1	0	0	1	**3**	no
Pleural fibrosis	0	0	0	1	1	1	**3**	no
**Caffeine**								
Increase of catecholamine levels	0	1	1	0	1	1	**4**	no
Interactions with uric acid metabolism	0	0	0	0	0	0	**0**	no
Tremor	1	1	1	0	1	1	**5**	yes
Seizures	1	1	0	1	1	1	**5**	yes
Anxiety	0	1	0	0	0	1	**2**	no
Increased gastric acid production	1	1	1	0	0	1	**4**	no
**Candesartan**								
Long duration of action	1	1	0	0	1	0	**3**	no
**Carbamazepine**								
Use-dependent block of neuronal discharges	1	0	1	1	0	1	**4**	no
**Cefepime**								
IND: Pseudomande infections	1	1	1	1	1	1	**6**	yes
IND: infections with gram negative bacteria	1	1	1	1	1	1	**6**	yes
IND: infections with gram positive bacteria	1	1	0	1	1	1	**5**	yes
**Ciclosporin**								
IND: autoimmune disorders, rheumatoide arthritis	1	0	1	1	1	1	**5**	yes
Variable oral bioavailability	1	1	1	1	0	1	**5**	yes
Predominant hepatic biotransformation	1	1	0	1	1	1	**5**	yes
Predominant hepatic elimination	0	0	0	0	0	1	**1**	no
Hyperglycemia	0	0	0	1	0	1	**2**	no
**Ciprofloxacin**								
Inhibition of topoisomerase II	1	1	1	0	0	1	**4**	no
Renal elimination	1	0	0	0	1	1	**3**	no
CNS adverse effects	1	0	0	0	1	0	**2**	no
**Clarithromycin**								
Alternative antibiotic for patients with penicillin allergy	1	1	0	1	0	1	**4**	no
**Cyclophosphamide**								
Immunsuppressive effect via inhibition of leucocytes	1	0	1	1	1	1	**5**	yes
**Diazepam**								
Dose-dependent anxiolytic, sedative, myotonolytic or anti-convultant	1	1	1	1	1	1	**6**	yes
conjugation of hydroxyl group during biotransformation	0	0	0	0	0	0	**0**	no
Reduced / slowed elimination in liver disease	1	0	1	1	0	1	**4**	no
Floppy infant syndrom	1	1	0	1	1	1	**5**	yes
Reduced libido	0	0	0	0	0	1	**1**	no
**Domperidone**								
IND: migraine attacks	0	1	0	0	1	1	**3**	no
Reflux	1	0	0	0	1	0	**2**	no
**Doxazosin**								
Tachycardia	1	1	0	0	1	1	**4**	no
Increased heart failure incidence	0	1	1	1	1	1	**5**	yes

**Doxycycline**								
IND: Proprioni bacteria infections	0	1	0	1	0	1	**3**	no
IND: Yersinia infections	0	0	0	1	0	1	**2**	no
IND: Protozoa infections	0	0	0	1	0	1	**2**	no
IND: Lymphgranuloma venerum	0	0	0	0	0	1	**1**	no
Penetrates blood-placenta-barrier	0	1	0	1	1	1	**4**	no
Variable penetration of blood-brain-barrier	0	0	0	0	0	1	**1**	no
Plasma protein binding 90%	0	0	0	0	0	0	**0**	no
Onycholysis	0	0	0	0	0	1	**1**	no
Increase of intracranial pressure	0	0	0	0	0	1	**1**	no
Esophageal mucositis	0	1	0	1	0	0	**2**	no
**Estradiol**								
IND: hormone therapy in menopause	1	1	1	1	1	1	**6**	yes
Binding to estradiol response element	1	0	0	1	1	1	**4**	no
Suppression of ovulation	1	1	0	1	1	1	**5**	yes
Higher oral dose	0	1	0	0	0	0	**1**	no
Hepatic elimination	1	1	0	1	1	0	**4**	no
Elimination product estriol	0	0	0	0	0	1	**1**	no
Glucuronidation, sulphation	0	1	0	1	0	1	**3**	no
Role of renal elimination	1	1	0	0	0	1	**3**	no
Transdermal application	1	1	1	1	1	1	**6**	yes
Thrombosis	1	1	1	1	1	1	**6**	yes
Endometrial carcinoma	1	1	1	1	1		**5**	yes
Edema	1	1	1	1	0	1	**5**	yes
Cardiovascular complications	1	1	1	1	1	1	**6**	yes
**Ethanol**								
Increase in blood pressure	0	1	0	0	1	0	**2**	no
Hypoglycemia	1	1	1	0	1	0	**4**	no
Increased diuresis	1	1	0	1	0	1	**4**	no
Decreased sexual potency	1	1	0	0	0	1	**3**	no
Distribution in total body water	0	1	0	1	1	1	**4**	no
Elimination increased in alcoholics	1	1	0	0	0	1	**3**	no
CYP450 metabolism	0	1	0	1	0	0	**2**	no
Energy source	1	1	0	1	1	1	**5**	yes
**Flucloxacillin**								
Effectiveness against penicilline-sensitive bacteria	0	1	0	1	1	1	**4**	no
Oral application	1	1	0	1	1	1	**5**	yes
Half-life 30 bis 60 min	0	0	0	0	0	0	**0**	no
High plasma protein binding	0	0	0	0	0	0	**0**	no
**Fluconazole**								
High water solubility	0	1	0	0	0	0	**1**	no
Application oral or i.v.	1	1	0	1	1	1	**5**	yes
Oral bioavailability > 90%	0	1	0	1	0	1	**3**	no
High liquor concentration	1	1	0	1	1	1	**5**	yes
Halflife 30 h	1	1	0	1	0	0	**3**	no
Mainly unmodified renal elimination	1	1	0	0	1	1	**4**	no
Dose adaption in renal insufficiency	1	1	1	1	1	1	**6**	yes
**Flumazenil**								
Half-life 1 h	1	1	0	0	0	0	**2**	no
**Furosemide**								
Effective if GFR <30 ml/min	1	1	1	1	1	1	**6**	yes
Increased thrombosis risk	1	1	1	1	1	1	**6**	yes
**Gentamicin**								
Intracelluar site of action	0	0	0	0	0	1	**1**	no
No penetration of blood-liquor-barrier	1	1	1	0		1	**4**	no
Low plasma protein binding	0	1	0	0	0	0	**1**	no
No metabolisation	1	1	0	0	0	1	**3**	no
Renal elimination by glomerular filtration	1	1	0	1	1	1	**5**	yes
Reduced elimination in renal insufficiency	1	1	1	1	1	1	**6**	yes
CI: pregnancy	1	1	0	1		1	**4**	no
**Glyceryl trinitrate**								
Duration of action 15-30 min	1	1	1	0	1	1	**5**	yes
**Goserelin**								
Regulation of GnRH-receptors	0	1	0	1	1	1	**4**	no
Reduction of sex hormone levels	1	1	1	1	1	1	**6**	yes
Hot flush	1	1	1	1	1	1	**6**	yes
**Heparin, unfractionated**								
Tissue irritation at injection site	0	0	0	0	0	1	**1**	no
**Hydrochlorothiazide**								
Hypercalciuria	0	1	0	1	1	1	**4**	no
Onset of action after 1-2 h	0	1	0	0	1	0	**2**	no
Loss of action if renal function is reduced	0	1	1	1	1	1	**5**	yes
**Ibuprofen**								
Oxidation as biotransformation process	0	0	0	0	0	0	**0**	no
**Imipenem**								
Beta-lactamase stable	1	1	1	1	1	1	**6**	yes
**Insulin lispro**								
Duration of action 2 h	1	1	0	0	1	1	**4**	no
Max. drug action after 40 min	1	1	0	0	1	1	**4**	no

**Isoflurane**								
MAC-value 1.2%	0	1	0	0	1	0	**2**	no
Metabolisation 0.2%	0	1	0	1	0	1	**3**	no
Quick onset of drug action	1	1	0	0	1	1	**4**	no
**Isoniazid**								
High tissue distribution	1	1	0	1	1	1	**5**	yes
**Lamotrigine**								
IND: focal seizures	1	1	1	1	0	1	**5**	yes
IND: Lennox-Gastaut-syndrome	0	0	0	1	1	0	**2**	no
**Levodopa**								
Metablization to dopamin by dopa-decarboxylase	1	1	1	1	1	1	**6**	yes
Loss of efficacy after longtime use	1	1	0	1	1	1	**5**	yes
**Lithium**								
Hyperthyreotic metabolic state	0	1	0	0	0	1	**2**	no
Therapeutic effect after 1-2 weeks	1	1	1	1	1	1	**6**	yes
Prophylactic effects after 6-12 months	1	1	1	1	1	1	**6**	yes
Maximal plasma concentration after 2 h	0	1	0	0	0	0	**1**	no
Euthyreot struma	1	1	1	1	1	1	**6**	yes
ECG abnormalities	1	1	0	1	1	1	**5**	yes
Renal diabetes insipidus	1	1	0	1	1	1	**5**	yes
**Loperamide**								
Inhibition of propulsive peristalis	1	1	1	1	1	1	**6**	yes
Reduction of fluid loss	1	1	1	1	1	1	**6**	yes
CNS AE in children	1	1	1	1	1	1	**6**	yes
**Metformin**								
Unmodified renal elimination	1	1	1	0	1	1	**5**	yes
Half-life 2-5 h	0	1	0	0	0	0	**1**	no
Caution if used in elderly patients	1	1	1	1	0	0	**4**	no
**Methotrexate**								
Direct inhibtion of enzymes	1	1	0	0	1	0	**3**	no
Drug resistance	0	1	0	0	1	0	**2**	no
**Metoclopramide**								
Antiemetic effect	1	1	1	1	1	1	**6**	yes
Increased tonus of lower esophagus sphincter	1	1	0	1	1	1	**5**	yes
Higher risk for AE in children	1	1	0	1	1	0	**4**	no
**Metoprolol**								
Blood pressure reducation after weeks	1	1	0	0	1	1	**4**	no
**Metronidazole**								
HP triple therapy	1	1	1	0	0	0	**3**	no
**Mirtazapine**								
Agitated depression	1	0	0	0	0	0	**1**	no
**Morphine**								
Sedation	1	1	1	1	1	1	**6**	yes
Oral bioavailability 15%	1	1	0	1	1	1	**5**	yes
Hepatic glucuronidation	1	0	0	0	0	0	**1**	no
Half-life: several hours	1	0	1	0	0	1	**3**	no
AE: increased GI-tonus	1	1	1	1	1	1	**6**	yes
AE: bradycardia	0	1	0	1	1	1	**4**	no
AE: miosis	1	1	0	1	1	1	**5**	yes
AE: effect on hypophyseal hormones	0	0	0	1	1	1	**3**	no
AE: delayed gastric emptying	0	1	0	1	1	1	**4**	no
AE: spastic constipation	1	1	1	1	1	1	**6**	yes
CI: lung diseases	0	0	0	0	1	0	**1**	no
Use during birth	1	0	0	1	1	1	**4**	no
**Naloxone**								
Use in neonatal medicine	0	1	0	0	1	0	**2**	no
Half-life approx. 1 h	1	1	1	0	1	0	**4**	no
**Nifedipine**								
Rapid onset of action	1	1	1	1	1	1	**6**	yes
Oral bioavailability 50%	0	1	0	0	0	0	**1**	no
Half-life 2 h	1	1	0	0	0	0	**2**	no
**Nicotine**								
Intoxication: excitation, followed by depolarisation block	1	1	0	1	1	1	**5**	yes
Absorption pH-dependent	0	1	0	1	0	0	**2**	no
Elimination: 10% unmodified renal elimination	0	1	0	1	0	0	**2**	no
Increase of plasma lipids	1	0	0	1	1	1	**4**	no
GI-ulcera	1	0	0	0	1	1	**3**	no
**Norepinephrine**								
α2-mediated inhibition of noradrenalin release	1	1	1	0	1	1	**5**	yes
Effect on β2 receptors	0	1	0	1	1	1	**4**	no
Renale elimination	1	1	0	0	0	1	**3**	no
**Omeprazole**								
Duration of action until re-sysnthesis of proton pump	1	1	1	0	1	1	**5**	yes
Duration of action > half-time (irreversible inhibition of proton pump)	1	1	0	0	1	1	**4**	no
Hypergastrinemia	1	0	0	0	1	0	**2**	no

**Ondansetrone**								
Co-medication with steroids results in increased efficacy	0	1	1	1	1	1	**5**	yes
Halflife 3.5 h	0	0	0	0	0	0	**0**	no
Duration serveral hours	0	0	0	1	1	1	**3**	no
**Pancuronium**								
IND: intoxications with elevated muscle tension	1	0	0	0	1	1	**3**	no
**Perchlorate**								
Aplastic anemia	1	1	1	1	1	0	**5**	yes
**Physostigmine**								
Carbamylation of ACh-esterase	0	0	1	0	0	0	**1**	no
**Pilocarpine**								
Accomodation errors	1	1	0	1	1	1	**5**	yes
Reduced twilight vision	0	1	0	1	1	1	**4**	no
Systemic actions	0	0	0	0	1	1	**2**	no
**Prednisolone**								
IND: leucemia	1	1	1	1	1	0	**5**	yes
Decreased cytokine synthesis	1	1	1	1	1	1	**6**	yes
Inhibition of all inflammation phases	1	0	1	1	1	1	**5**	yes
Negative feedback mechanisms	1	1	1	1	1	1	**6**	yes
Affinity to mineral corticoid receptor	1	1	1	1	1	1	**6**	yes
Oral application	0	1	0	0	1	1	**3**	no
Glucuronidation, sulphatation	0	1	0	0	0	0	**1**	no
Sodium retention, potassium excretion via mineral corticoid receptor action	1	1	1	0	1	1	**5**	yes
Muscle weakness	1	1	0	1	1	1	**5**	yes
Euphoria	1	1	1	1	1	1	**6**	yes
Adrenal cortex atrophia	1	1	1	1	1	1	**6**	yes
Glaucoma, cataract	0	1	1	1	1	1	**5**	yes
CI: osteoporosis	1	1	1	1	1	1	**6**	yes
CI: GI-ulcera	1	1	1	1	1	1	**6**	yes
CI: hypertonus	1	1	1	0	1	1	**5**	yes
CI: diabetes mellitus	1	1	0	1	1	1	**5**	yes
CI: infectious diseases	1	1	1	1	1	1	**6**	yes
CI: pregnancy	1	1	0	1	1	1	**5**	yes
**Propofol**								
Drug action after 1 min	0	1	0	0	1	1	**3**	no
Duration of action 10 min	1	1	0	0		1	**3**	no
Half-time 30 min	0	1	0	0		0	**1**	no
Hepatic metabolisation	1	1	0	0	0	1	**3**	no
**Pyrazinamide**								
Inhibition of fatty acid synthesis by pyrazincarbone acid	0	0	0	0	0	0	**0**	no
Inhibition of mycolic acid synthesis	1	1	1	1	1	1	**6**	yes
High tissue distribution	0	1	0	0		1	**2**	no
**Ranitidine**								
Dizziness	1	0	0	0	1	1	**3**	no
**Rifampicin**								
Application p.o.	0	1	0	0	0	1	**2**	no
Plasma protein binding 80%	0	1	0	0	0	0	**1**	no
High organ distribution	1	1	0	1	0	1	**4**	no
Half-life 2-5 h	0	1	0	0	0	0	**1**	no
After longtime use reduction of half-life	0	1	0	1	1	1	**4**	no
GI-tract side effects	0	1	0	0	1	1	**3**	no
Adverse effects of the skin	1	1	0	0	1	1	**4**	no
Neurological side effects	1	0	0	0	0	1	**2**	no
Flu symptoms	0	1	0	0	1	1	**3**	no
Use in pregnancy	0	1	0	0	1	1	**3**	no
Use during lactation	0	1	0	0	1	1	**3**	no
**Suxamethonium**								
Blockade of action potentials	1	1	0	0	1	1	**4**	no
Longlasting paralysis	1	1	0	1	1	0	**4**	no
**Tazobactam**								
IND: Severe infections	0	1	0	0	1	1	**3**	no
**Thiamazole (Methimazol)**								
Inhibition of iodine incorporation to tyrosines	1	1	1	0	1	1	**5**	yes
Delayed thyreostatic effect	1	1	1	1	1	1	**6**	yes
**Valproic acid**								
IND: focal seizures	1	0	1	1	0	1	**4**	no
Inhibition of T-type-calcium channels	0	0	0	0	0	1	**1**	no
High plasma protein binding	1	1	0	1	0	0	**3**	no
**Vancomycin**								
No penetration of outer membrane in gram negative bacteria	0	0	1	1	1	1	**4**	no
No hemodialysis	0	0	0	1	0	0	**1**	no
Irritation of veins after i.v. application	0	1	0	1	1	1	**4**	no
