# Supplementary material for: Accuracy and Completeness of Drug Information in Wikipedia: A Comparison with Standard Textbooks of Pharmacology
Source: PLoS One. 2014 Sep 24;9(9):e106930. doi: 10.1371/journal.pone.0106930 (PMC4174509; doi:10.1371/journal.pone.0106930)
Supplement: Table S4 — Proportion of references per drug article in Wikipedia. (PDF) [file pone.0106930.s004.pdf]

Table S4: Proportion of references per drug article in Wikipedia.

German Wikipedia	A	B	C	D	E	F	G
Acetylsalicylic acid	0.42	0.22	0.05	0.07	0.00	0.13	0.11
Aciclovir	0.18	0.45	0.00	0.00	0.00	0.27	0.09
Alendronic acid	0.00	0.00	0.00	0.00	0.00	1.00	0.00
Allopurinol	0.11	0.00	0.11	0.00	0.00	0.78	0.00
Amantadine	0.17	0.17	0.08	0.08	0.00	0.33	0.17
Amiodarone	0.33	0.33	0.05	0.00	0.00	0.29	0.00
Amitriptyline	0.44	0.06	0.13	0.00	0.00	0.38	0.00
Amoxicillin	0.00	0.00	0.00	0.50	0.00	0.50	0.00
Atropine	0.33	0.33	0.00	0.00	0.00	0.33	0.00
Azathioprine	0.08	0.17	0.00	0.08	0.00	0.42	0.25
Benzylpenicillin	0.08	0.38	0.00	0.00	0.00	0.54	0.00
Biperiden	0.20	0.40	0.00	0.00	0.00	0.40	0.00
Bromocriptine	0.20	0.20	0.00	0.00	0.00	0.60	0.00
Buprenorphine	0.58	0.22	0.03	0.00	0.00	0.11	0.06
Caffeine	0.46	0.17	0.04	0.12	0.00	0.19	0.02
Candesartan	0.00	0.17	0.00	0.00	0.00	0.83	0.00
Carbamazepine	0.14	0.14	0.14	0.07	0.00	0.36	0.14
Cefepime	0.50	0.00	0.25	0.00	0.00	0.25	0.00
Ciclosporin	0.22	0.11	0.00	0.00	0.00	0.56	0.11
Ciprofloxacin	0.37	0.16	0.00	0.00	0.00	0.47	0.00
Clarithromycin	0.00	0.20	0.20	0.10	0.00	0.50	0.00
Clopidogrel	0.32	0.13	0.35	0.03	0.00	0.16	0.00
Cocaine	0.13	0.21	0.30	0.13	0.00	0.11	0.13
Cyclophosphamide	0.09	0.27	0.09	0.00	0.00	0.55	0.00
Diazepam	0.19	0.19	0.05	0.00	0.00	0.48	0.10
Digoxin	0.22	0.00	0.00	0.33	0.00	0.44	0.00
Domperidone	0.00	0.33	0.33	0.00	0.00	0.33	0.00
Doxazosin	0.00	0.40	0.00	0.00	0.00	0.60	0.00
Doxycycline	0.00	0.40	0.10	0.00	0.00	0.50	0.00
Enoxaparin sodium	0.50	0.08	0.08	0.00	0.00	0.25	0.08
Epinephrine	0.20	0.30	0.20	0.00	0.00	0.30	0.00
Estradiol	0.17	0.17	0.00	0.17	0.00	0.50	0.00
Ethambutol	0.40	0.20	0.00	0.00	0.00	0.40	0.00
Ethanol	0.16	0.49	0.09	0.06	0.01	0.09	0.10
Exenatide	0.44	0.00	0.22	0.11	0.00	0.22	0.00
Finasteride	0.30	0.17	0.17	0.00	0.00	0.17	0.17
Flucloxacillin	0.33	0.67	0.00	0.00	0.00	0.00	0.00
Fluconazole	0.17	0.17	0.00	0.00	0.00	0.67	0.00
Flumazenil	0.20	0.40	0.00	0.00	0.00	0.40	0.00
Furosemide	0.20	0.00	0.60	0.00	0.00	0.00	0.20
Gentamicin	0.56	0.19	0.00	0.25	0.00	0.00	0.00
Glyceryl trinitrate	0.19	0.38	0.00	0.00	0.00	0.38	0.06

Goserelin	0.60	0.00	0.00	0.00	0.40	0.00	0.00
Haloperidol	0.38	0.15	0.00	0.00	0.00	0.38	0.08
Heparin	0.00	0.38	0.08	0.00	0.00	0.54	0.00
Hydrochlorothiazide	0.00	0.50	0.10	0.00	0.10	0.30	0.00
Ibuprofen	0.40	0.16	0.00	0.08	0.00	0.24	0.12
Imipenem	0.00	0.00	0.00	0.00	0.00	1.00	0.00
Insulin lispro	0.50	0.50	0.00	0.00	0.00	0.00	0.00
Isoflurane	0.00	0.50	0.00	0.00	0.00	0.50	0.00
Isoniazid	0.40	0.20	0.00	0.00	0.00	0.40	0.00
Lamotrigine	0.54	0.08	0.00	0.00	0.00	0.31	0.08
Levodopa	0.33	0.22	0.22	0.00	0.00	0.22	0.00
Lithium	0.33	0.27	0.13	0.00	0.00	0.20	0.07
Loperamide	0.14	0.29	0.00	0.00	0.00	0.57	0.00
Metamizole	0.31	0.21	0.21	0.10	0.00	0.10	0.07
Metformin	0.47	0.18	0.18	0.06	0.00	0.12	0.00
Methanol	0.13	0.40	0.20	0.02	0.06	0.04	0.14
Methimazole	0.00	0.00	0.00	0.00	0.00	1.00	0.00
Methotrexate	0.17	0.17	0.33	0.00	0.00	0.33	0.00
Methyldopa	0.00	0.40	0.00	0.00	0.00	0.60	0.00
Methylphenidate	0.24	0.24	0.24	0.07	0.02	0.13	0.07
Metoclopramide	0.09	0.27	0.27	0.00	0.00	0.36	0.00
Metoprolol	0.22	0.33	0.00	0.00	0.00	0.44	0.00
Metronidazole	0.00	0.50	0.00	0.00	0.00	0.50	0.00
Mifepristone	0.00	0.25	0.25	0.00	0.00	0.50	0.00
Mirtazapine	0.71	0.05	0.05	0.02	0.00	0.17	0.00
Molsidomine	0.00	0.22	0.00	0.00	0.22	0.56	0.00
Morphine	0.13	0.50	0.00	0.00	0.00	0.19	0.19
Naloxone	0.20	0.00	0.00	0.00	0.00	0.80	0.00
Nicotine	0.55	0.08	0.18	0.04	0.00	0.10	0.06
Nifedipine	0.07	0.29	0.07	0.00	0.00	0.36	0.21
Norepinephrine	0.13	0.25	0.00	0.00	0.00	0.63	0.00
Omeprazole	0.33	0.25	0.17	0.00	0.00	0.25	0.00
Ondansetron	0.33	0.17	0.17	0.00	0.00	0.33	0.00
Paclitaxel	0.44	0.04	0.20	0.00	0.08	0.24	0.00
Pancuronium	0.00	0.25	0.00	0.00	0.00	0.75	0.00
Perchlorate	0.40	0.40	0.20	0.00	0.00	0.00	0.00
Phenobarbital	0.42	0.25	0.08	0.00	0.00	0.25	0.00
Physostigmine	0.17	0.50	0.00	0.00	0.00	0.33	0.00
Pilocarpine	0.00	0.29	0.00	0.00	0.00	0.71	0.00
Piperacillin	0.14	0.43	0.00	0.00	0.00	0.43	0.00
Prednisolone	0.29	0.29	0.14	0.00	0.00	0.29	0.00
Propofol	0.53	0.21	0.00	0.11	0.00	0.16	0.00
Pyrazinamide	0.00	0.00	0.00	0.00	0.00	1.00	0.00
Ramipril	0.17	0.17	0.00	0.00	0.00	0.67	0.00
Ranitidine	0.50	0.10	0.00	0.00	0.00	0.30	0.10
Repaglinide	0.50	0.00	0.00	0.00	0.00	0.50	0.00
Rifampicin	0.00	0.14	0.00	0.14	0.00	0.71	0.00
Rituximab	0.76	0.00	0.19	0.00	0.00	0.05	0.00

Rivaroxaban	0.33	0.11	0.22	0.22	0.06	0.00	0.06
Sitagliptin	0.33	0.00	0.33	0.00	0.00	0.33	0.00
Somatotropin	0.38	0.08	0.15	0.15	0.00	0.15	0.08
Spironolactone	0.33	0.50	0.00	0.00	0.00	0.17	0.00
Suxamethonium chloride	0.00	1.00	0.00	0.00	0.00	0.00	0.00
Tamoxifen	0.44	0.13	0.13	0.00	0.00	0.25	0.06
Tazobactam	0.00	0.25	0.00	0.00	0.00	0.75	0.00
Tramadol	0.56	0.28	0.06	0.00	0.00	0.11	0.00
Valproic acid	0.31	0.13	0.25	0.00	0.00	0.25	0.06
Vancomycin	0.23	0.31	0.31	0.08	0.00	0.00	0.08

**English Wikipedia**	**A**	**B**	**C**	**D**	**E**	**F**	**G**
Aciclovir	0.57	0.17	0.09	0.04	0.00	0.13	0.00
Allopurinol	0.85	0.04	0.00	0.04	0.00	0.08	0.00
Amantadine	0.66	0.07	0.07	0.07	0.03	0.03	0.07
Atropine	0.21	0.50	0.07	0.00	0.00	0.07	0.14
Buprenorphine	0.55	0.05	0.12	0.01	0.05	0.05	0.15
Clopidogrel	0.44	0.06	0.31	0.03	0.06	0.06	0.06
Diazepam	0.65	0.06	0.03	0.03	0.03	0.04	0.17
Finasteride	0.59	0.01	0.12	0.12	0.04	0.04	0.07
Flumazenil	0.56	0.44	0.00	0.00	0.00	0.00	0.00
Ibuprofen	0.56	0.02	0.15	0.11	0.03	0.05	0.09
Lamotrigine	0.67	0.06	0.08	0.00	0.05	0.05	0.11
Loperamide	0.35	0.10	0.00	0.00	0.00	0.40	0.15
Methorexate	0.71	0.10	0.00	0.05	0.00	0.05	0.10
Mirtazapine	0.81	0.08	0.00	0.02	0.00	0.05	0.04
Nifedipine	0.68	0.11	0.00	0.00	0.00	0.21	0.00
Paclitaxel	0.52	0.27	0.06	0.00	0.00	0.02	0.13
Pilocarpine	0.71	0.14	0.00	0.00	0.00	0.14	0.00
Propofol	0.59	0.04	0.02	0.22	0.02	0.00	0.12
Rituximab	0.66	0.03	0.09	0.03	0.06	0.03	0.11
Vancomycin	0.79	0.12	0.04	0.02	0.02	0.00	0.02

Legend:

A: academic publications
B: textbooks
C: professional organizations
D: news media
E: commerical companies
F: databases/ prescription info
G: others
