# Supplementary material for: Accuracy and Completeness of Drug Information in Wikipedia: A Comparison with Standard Textbooks of Pharmacology
Source: PLoS One. 2014 Sep 24;9(9):e106930. doi: 10.1371/journal.pone.0106930 (PMC4174509; doi:10.1371/journal.pone.0106930)
Supplement: Table S5 — Readability scores for German Wikipedia. (PDF) [file pone.0106930.s005.pdf]

Table S5: Readability scores for German Wikipedia.

**Amstad**	Textbooks	Wikipedia
Aciclovir	9.75	10.96
Acetylsalicylic acid	0.99	8.00
Azathioprine	1.16	6.15
Clopidogrel	4.21	3.40
Metronidazole	6.54	1.81
Morphine	18.33	15.82
Nifedipine	3.89	3.95
Propofol	12.89	1.03
Spironolacton	4.53	12.64

**1. WSTF**	Textbooks	Wikipedia
Aciclovir	14.58	14.33
Acetylsalicylic acid	14.35	13.98
Azathioprine	14.87	14.94
Clopidogrel	15.08	15.80
Metronidazole	14.60	16.46
Morphine	13.20	14.61
Nifedipine	14.95	17.10
Propofol	13.50	15.87
Spironolacton	14.81	13.46
