# Supplementary material for: Accuracy and Completeness of Drug Information in Wikipedia: A Comparison with Standard Textbooks of Pharmacology
Source: PLoS One. 2014 Sep 24;9(9):e106930. doi: 10.1371/journal.pone.0106930 (PMC4174509; doi:10.1371/journal.pone.0106930)
Supplement: Table S6 — Number of edits and editors per drug article. (PDF) [file pone.0106930.s006.pdf]

Table S6: Number of edits and editors per drug article.

German Wikipedia	edits	editors		
		registred	anonymous	bots
Acetylsalicylic acid	206	63	50	12
Aciclovir	208	63	42	20
Allopurinol	118	34	24	18
Amantadine	143	46	20	18
Amiodaron	193	50	35	15
Amitriptyline	216	77	33	20
Amoxicillin	184	59	34	27
Atropine	465	147	111	35
Azathioprine	178	50	29	19
Benzylpenicillin	2	2	0	0
Biperiden	98	31	11	17
Bromocriptine	66	22	9	12
Buprenorphine	368	71	77	17
Caffeine	1194	347	311	44
Candesartan	82	29	3	11
Carbamazepine	160	61	24	19
Ciclosporin	135	46	17	18
Ciprofloxacin	223	67	36	19
Clarithromycin	90	28	19	17
Clopidogrel	300	67	40	22
Cocaine	1405	366	354	52
Cyclophosphamide	154	49	28	24
Diazepam	530	147	138	24
Digoxin	90	28	9	16
Domperidon	97	37	16	14
Doxazosin	59	19	0	9
Doxycycline	161	53	24	22
Enoxaparin sodium	177	30	4	7
Epinephrine	660	186	136	35
Estradiol	151	49	18	20
Ethambutol	66	33	4	13
Ethanol	2066	501	310	55
Exenatide	111	35	12	7
Finasteride	304	81	84	18
Flucloxacillin	33	9	2	3
Fluconazole	65	21	3	11
Flumazenil	87	26	12	14
Furosemide	114	47	20	19
Gentamicin	179	46	25	22
Glyceryl trinitrate	3	2	0	0
Haloperidol	298	80	67	22
Hydrochlorothiazide	81	35	10	17
Ibuprofen	572	157	149	31
Imipenem	62	24	6	12

Isoflurane	62	23	6	15
Isoniazid	80	33	13	17
Lamotrigine	195	54	39	19
Levodopa	152	58	34	22
Lithium	1381	320	247	59
Loperamide	133	48	31	13
Metamizole	306	74	61	21
Metformin	202	59	40	21
Methanol	1383	271	258	37
Methotrexate	175	49	55	22
Methyldopa	85	24	6	15
Methylphenidate	1418	277	343	17
Metoclopramide	158	61	24	17
Metoprolol	201	52	34	16
Metronidazol	163	50	30	22
Mifepriston	165	78	27	18
Mirtazapine	288	76	59	15
Molsidomine	55	19	4	10
Morphine	650	195	174	34
Naloxone	156	59	17	19
Nicotine	939	255	202	39
Nifedipine	114	39	26	12
Norepinephrine	240	80	58	24
Omeprazole	218	61	43	23
Ondansetron	86	32	5	12
Paclitaxel	194	54	29	23
Pancuronium	73	22	11	17
Perchlorate	1	1	0	0
Phenobarbital	187	70	28	21
Physostigmine	122	32	18	16
Pilocarpine	65	20	8	14
Piperacillin	53	19	1	12
Prednisolone	133	42	31	10
Propofol	311	88	54	23
Pyrazinamide	56	18	6	15
Ramipril	116	37	25	15
Ranitidine	152	43	18	20
Rifampicin	110	34	15	20
Rituximab	139	51	21	15
Rivaroxaban	129	43	15	13
Sitagliptin	140	34	15	12
Somatropin	320	87	77	26
Spironolacton	99	27	16	19
Suxamethonium	70	26	4	15
Tamoxifen	143	45	15	12
Tazobactam	31	12	0	9
Thiamazole	55	14	9	7
Tramadol	442	110	128	22
Vancomycin	141	53	21	17
